# Electrochemical Dopamine Biosensor Based on Plant-Derived Peroxidase Immobilized on Titanate Nanowires

**DOI:** 10.1021/acsomega.6c02913

**Published:** 2026-06-17

**Authors:** Daniel Ananias Reis de Campos, Guilherme Sales da Rocha, Neuman Solange de Resende, Helen Conceição Ferraz, Inês Rosane Welter Zwirtes de Oliveira, João Victor Nicolini

**Affiliations:** † Universidade Federal do Rio de Janeiro, Rio de Janeiro, State of Rio de Janeiro 21941- 853, Brazil; ‡ Universidade Federal Rural do Rio de Janeiro, Seropédica, State of Rio de Janeiro 23890-000, Brazil

## Abstract

Dysregulation of dopamine (DA) levels is strongly associated with a range of neurodegenerative and psychiatric disorders, including Parkinson’s disease. Limitations inherent to conventional analytical techniques have prompted the development of alternative methods, particularly electrochemical biosensors, which provide rapid response and real-time analysis. Among enzyme-based biosensors, peroxidases are notable for their catalytic efficiency and versatility. In this study, gherkin (*Cucumis anguria* L.) extract was immobilized onto titanate nanowires (TNWs) to fabricate four electrochemical biosensors for DA detection. Calibration curves generated by square-wave voltammetry demonstrated a linear range of 5–65.4 μmol L^–1^ for all biosensors. The most effective biosensor (TNW-C) exhibited the equation *I*_pa_ = 0.573_DA_ + 2.44 (*R* ^2^ = 0.999), with a limit of detection of 0.400 μmol L^–1^, a limit of quantification of 1.21 μmol L^–1^, and a sensitivity of 8.19 μA μmol L^–1^ cm^–2^. Application of TNW-C to a commercial sample resulted in a DA concentration of 4.99 ± 0.40 mg mL^–1^, corresponding to a relative error of −0.2% compared to the labeled value of 5 mg mL^–1^. Evaluation of urea as a potential interferent revealed only a slight current decrease (−0.9%). Additional assays with uric acid showed well-resolved oxidation peaks, with the DA current for TNW-C being 3.68 times higher than that of uric acid. The response also agreed with UV–vis analysis and the labeled pharmaceutical value, supporting real-sample applicability. These results demonstrate that peroxidase-rich gherkin extract is an effective and potentially cost-effective biocatalyst for DA biosensing, serving as a promising alternative to commercial horseradish peroxidase in nanobiocomposite sensing platforms.

## Graphical abstract

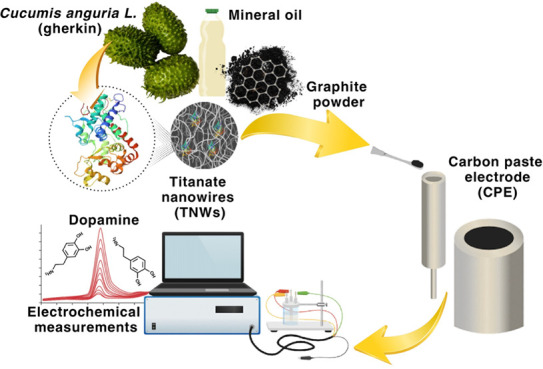

## 1 Introduction

Dopamine (DA) (3,4-dihydroxyphenylethylamine) is a key catecholamine neurotransmitter that regulates a range of physiological functions, including motor activity, reward-motivated behavior, cognition, memory, and endocrine modulation.^1,2^ As a chemical messenger in both the central nervous system and peripheral tissues, DA modulates neural communication and systemic physiological responses.^3^ Dysregulation of DA levels is closely associated with neurodegenerative and psychiatric disorders, such as Parkinson’s disease, Alzheimer’s disease, schizophrenia, attention deficit hyperactivity disorder (ADHD), and addiction-related conditions.^1,4–6^

Normal DA plasma concentrations range from 0.1 to 1.0 μmol L^–1^,^7^ while urinary DA levels typically fall between 0.1 and 4.7 μmol L^–1^.^8^ Deviations from these ranges may be linked to the aforementioned disorders.^9^ Consequently, the development of analytical methods with high sensitivity and selectivity for DA detection is critical, particularly in complex biological matrices that contain interfering species, such as ascorbic acid, uric acid, and other electroactive neurotransmitters.^10^

Conventional DA detection methods, such as high-performance liquid chromatography (HPLC),^11^ capillary electrophoresis,^12^ chemiluminescence,^13^ and spectrophotometric techniques,^14^ are accurate and widely used. However, these approaches often require expensive instrumentation, complex sample preparation, and lengthy analysis times. Chromatographic and electrophoretic methods are limited by their need for centralized laboratory infrastructure. In comparison, spectrophotometric methods lack selectivity in complex samples due to overlapping signals and interference. Unlike these, chemiluminescence-based methods require tightly regulated reaction conditions and use specialized detectors. These distinct limitations^15^ collectively reduce the suitability of all three approaches for rapid, portable, and point-of-care DA analysis.

Electrochemical techniques have emerged as rapid and straightforward alternatives for analyzing redox-active molecules, making them promising for neurotransmitter detection, including DA. Rapid real-time measurement of DA is essential for the early diagnosis of DA-related neurological disorders and has attracted increasing research interest.^16^ The limitations of traditional methods have prompted the development of alternative strategies, particularly electrochemical biosensors, which offer advantages such as fast response, operational simplicity, portability, and real-time, in situ analysis capabilities.^17,18^ These biosensors typically employ biomolecules, including enzymes,^19^ antibodies,^20^ or aptamers,^21^ as recognition elements for selective DA detection. Among transduction strategies, enzyme-based electrochemical biosensors are distinguished by their specificity and catalytic amplification, enabling highly sensitive detection at trace concentrations.

Peroxidases are widely used in biosensing platforms due to their ability to catalyze redox reactions involving hydrogen peroxide (H_2_O_2_)^22,23^ and their responsiveness to a broad range of analytes, including phenolic compounds such as DA.^24^ The enzymatic oxidation of DA in the presence of H_2_O_2_ produces electroactive species,^25^ which can be quantified using voltammetric or amperometric techniques. However, horseradish peroxidase (HRP), the primary commercially available peroxidase, has notable limitations, including high cost, limited long-term stability, and susceptibility to denaturation.^26^ These challenges underscore the need for alternative, low-cost, and environmentally sustainable sources of peroxidase-like activity.

Despite the extensive literature on enzyme-based electrochemical biosensors, most reported systems still rely on purified commercial enzymes, which increases cost and limits sustainability. In this context, relatively few studies have systematically evaluated whether crude or semipurified plant extracts, when properly immobilized onto nanostructured supports, can achieve analytical performance comparable to that of purified enzymes. Plant extracts have therefore emerged as promising candidates due to their inherent enzymatic content, ease of extraction, biodegradability, and alignment with green chemistry principles.^27,28^ Among these, *Cucumis anguria* L., commonly known as gherkin, is a cucurbitaceous plant native to tropical and subtropical regions, particularly in South America and Africa. Traditionally used in local medicine and cuisine, recent studies have identified peroxidase-like enzymes in its crude extracts.^29^ These natural biocatalysts are viable alternatives to conventional enzyme sources in biosensor applications, especially when integrated with effective immobilization strategies and nanostructured platforms.^30^

Effective enzyme immobilization is essential for ensuring the operational stability, reusability, and overall performance of biosensors.^31^ Nanostructured materials are well-suited for this purpose due to their high surface-to-volume ratio, tunable morphology, and superior electron-transport properties.^32,33^ Titanate nanowires (TNWs), as one-dimensional nanomaterials, offer a large specific surface, high ion-exchange capacity, and excellent biocompatibility, facilitating robust enzyme attachment and preserving catalytic activity. Additionally, TNWs exhibit favorable electrochemical properties that enhance signal transduction and improve the analytical sensitivity of biosensors.^34–36^

This study introduces a novel biosensor in which gherkin (*Cucumis anguria* L.) extract, serving as an enzymatic source, is immobilized on TNWs for the electrochemical detection of DA. The key innovation lies in the direct integration of plant-derived peroxidase with TNWs, forming a unique bioelectrocatalytic interface for DA sensing—a combination not previously reported. Unlike typical biosensors that rely on purified commercial enzymes like HRP, this approach demonstrates that carefully processed plant extracts can be incorporated into nanostructured platforms while retaining high catalytic activity and analytical performance. This integration not only offers a natural, low-cost enzymatic alternative but also advances sustainable, environmentally friendly nanobiocomposite biosensors for analytical and biomedical monitoring.

## 2 Experimental Section

### 2.1 Materials and Reagents

Guaiacol (99%, Spectrum), graphite powder (99.99%, Acheson 38, Thermo Fisher Scientific), hydrogen peroxide (30%, Vetec), glutaraldehyde (25%, Vetec), sodium bicarbonate (99%, Isofar) and sodium carbonate (99%, Isofar). Acetic acid (99.7%), sodium phosphate monobasic (99%) and sodium phosphate dibasic (99%) were purchased from Tedia (Fairfield, USA). 3-Aminopropyltrimethoxysilane (APTMS) (99%), dopamine hydrochloride (98%), mineral oil, sodium acetate (99%), and uric acid (99%) were purchased from Sigma-Aldrich (St. Louis, USA). Phosphate buffer saline solution (PBS, 0.1 mol L^–1^) was prepared by dissolving sodium phosphate monobasic and sodium phosphate dibasic (Tedia) in distilled water, and the pH was adjusted to 6, 7, or 8 as required. Gherkin (*Cucumis anguria* L.) homogenate served as the peroxidase source and was obtained from a local market in Rio de Janeiro, Brazil.

### 2.2 Functionalization of TNWs

Protonated titanate nanowires (TNWs, H_2_Ti_3_O_7_) were synthesized at the Laboratory of Interface Phenomena Engineering, Federal University of Rio de Janeiro (LABEFIT, UFRJ), and supplied for this study. The synthesis involved alkaline hydrothermal treatment of titanium dioxide (TiO_2_) powder with 10 mol L^–1^ sodium hydroxide (NaOH) solution, followed by proton exchange through rinsing with 1 mol L^–1^ nitric acid (HNO_3_) solution, as previously described.^34,35^ TNWs were functionalized with amino groups according to established protocols.^34,35^ Briefly, 100 mg of TNWs were dispersed in 10 mL of dichloromethane and sonicated for 20 min to obtain a homogeneous suspension. Subsequently, 0.1 mL of APTMS was added dropwise under continuous magnetic stirring and the mixture was stirred for 3 h at room temperature. The suspension was centrifuged for 2 min, and the supernatant was discarded. The resulting solid was washed with 5 mL of dichloromethane and centrifuged; this washing step was repeated twice to remove unreacted reagents. The final solid was dried at 60 °C for 10 min. APTMS-functionalized TNWs (TNW-APTMS, 100 mg) were resuspended in 5 mL of a 2.5% (w/w) glutaraldehyde (GA) solution prepared in 0.1 mol L^–1^ PBS (pH 7) and stirred for 1 h. The suspension was centrifuged for 2 min, the supernatant was discarded and the pellet was washed with 30 mL of PBS (pH 7). This washing step was repeated twice. The material was then dried at 60 °C for 30 min. Subsequently, the resulting GA-functionalized TNWs (TNW-APTMS-GA) were used for peroxidase immobilization.

The chemical structure, surface functionalization, morphology and crystalline structure of TNWs were examined using X-ray diffraction analysis (XRD), Zeta potential (ζ), Fourier Transform Infrared (FTIR) spectroscopy, Scanning electron microscopy/Energy-dispersive X-ray spectroscopy (SEM/EDX) before and after functionalization. Zeta potential (ζ) measurements (Litesizer DLS, Anton Paar GmbH) were carried out to determine potential for each step of immobilization strategy. FTIR spectra were acquired with a Bruker Vertex 70 spectrophotometer in Attenuated Total Reflectance (ATR) mode, operated via OPUS software (version 6.5). Spectra were collected over a wavenumber range of 4000 to 500 cm^–1^ with a spectral resolution of 4 cm^–1^. This approach facilitated the identification of characteristic vibrational bands corresponding to the titanate structure and the functional groups introduced during surface modification, thereby enabling evaluation of successful functionalization and alterations in surface chemistry. The morphology and elemental mapping of TNWs and each immobilization step were analyzed using field-emission scanning electron microscopy (FEG-SEM) with EDX. A Tescan MIRA fourth generation LMU microscope (LowVac Mode UniVac, 1–700 Pa) with an EDX detector was used at 10 keV. Micrographs were taken at 50,000× and 100,000× magnifications. Crystallinity of the synthesized material was confirmed by XRD (Rigaku Ultima IV diffractometer) with copper Kα radiation (λ = 1.5418 Å) at 40 kV and 20 mA. Data were collected in continuous-scan mode from 5° to 80° (2θ), using 0.02° steps and a scan speed of 10° min^–1^.

### 2.3 Preparation of the Gherkin Tissue Homogenate

A peroxidase-rich extract was prepared by homogenizing 25 g of *Cucumis anguria* L. (gherkin) in 100 mL of 0.1 mol L^–1^ phosphate-buffered saline (PBS, pH 7.0) for 60 s using a high-speed homogenizer. The resulting mixture was filtered through cotton gauze to remove coarse fibrous material. The filtrate was then centrifuged at 4620 × *g* for 10 min at 4 °C, and the supernatant was collected. This supernatant was subsequently filtered through 0.45 and 0.22 μm membrane filters (Millipore) to remove remaining particulates, yielding a clarified enzymatic extract, hereafter referred to as POX. The extract was stored at 4 °C until further use (Figure 1).

**Figure 1:**
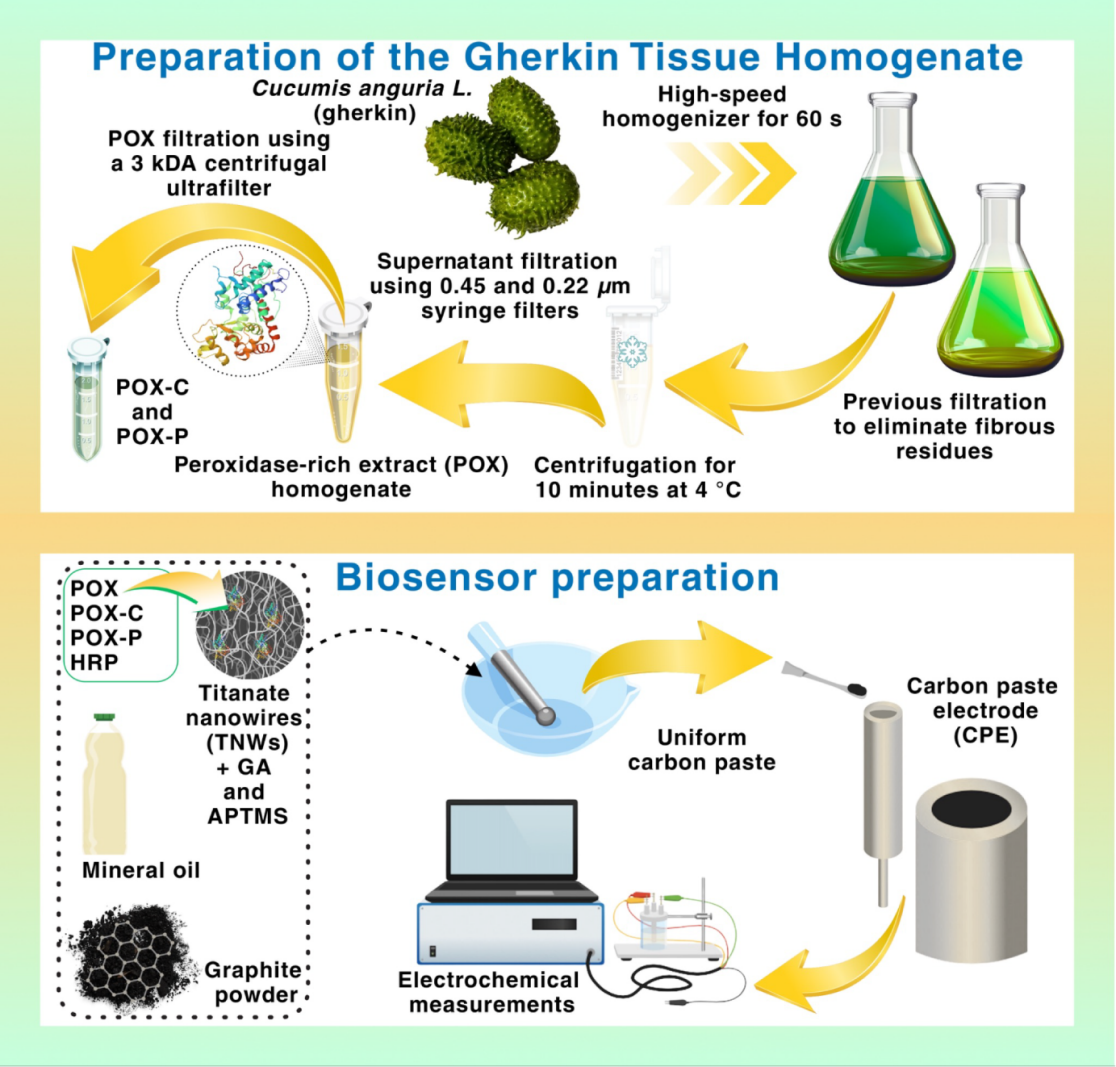
Schematic representation of preparation of homogenate from gherkin (*Cucumis anguria* L.) and biosensor preparation.

For fractionation, POX was subjected to centrifugal ultrafiltration using a Centriprep device with a molecular weight cutoff (MWCO) of 3 kDa. Briefly, 5 mL of the extract was loaded into the filtration unit and centrifuged at 3,000 × *g* for 50 min at approximately 25 °C using a swinging-bucket rotor, following the manufacturer’s guidelines. This procedure yielded two fractions: the retained fraction (POX-C), corresponding to the concentrate, and the permeate fraction (POX-P), corresponding to the filtrate. This step was performed to evaluate whether partial fractionation of the crude extract could influence enzymatic activity and biosensor performance.

### 2.4 pH Effect, Enzymatic Activity, Stability, and Molecular Mass Determination

Buffer solutions with pH values ranging from 4 to 9 were prepared at a final concentration of 0.1 mol L^–1^. Specifically, acetic acid and sodium acetate were used to prepare solutions at pH 4, 4.5, 5, and 5.5, while hydrogen phosphate and dihydrogen phosphate were used to prepare solutions at pH 6, 6.5, 6.8, 6.9, 7, 7.1, 7.2, and 7.5. For pH 8, 8.5, and 9, sodium bicarbonate and sodium carbonate were employed.

Peroxidase activity in *Cucumis anguria* L. extracts was assessed for the three homogenates: POX, POX-C, and POX-P. For comparison, the activity of commercial HRP was measured under identical experimental conditions. The assay consisted of mixing 0.2 mL of enzymatic extract with 2.7 mL of 50 mmol L^–1^ guaiacol solution and 0.1 mL of 10 mmol L^–1^ H_2_O_2_ solution, both prepared in 0.1 mol L^–1^ PBS (pH 7). The enzymatic activity was evaluated at 25 °C in triplicate by monitoring tetraguaiacol formation at 470 nm. One unit of enzymatic activity was defined as an increase of 0.001 in absorbance per minute at 470 nm. After preparation, the activities of the fractionated extracts (POX-C and POX-P) and the HRP solution were monitored for 4 weeks. The HRP solution was prepared at a concentration of 0.032 mg mL^–1^, and both the extracts and the HRP solution were stored at −6 °C throughout the analysis period.

Following buffer and enzyme solution preparation and storage, the approximate molecular mass of POX was determined by gel filtration (molecular exclusion chromatography). Three Sephadex silica gel matrices with distinct fractionation ranges were employed: G-25 (1–5 kDa), G-50 (1.5–30 kDa), and G-75 (3–80 kDa). Analyses were performed using a chromatographic column measuring 22 cm in height and 0.5 cm in diameter. The elution phase consisted of 0.1 mol L^–1^ PBS buffer adjusted to pH 7. A 0.2 mL volume of POX, exhibiting an enzymatic activity of 8,850 U mL^–1^, was applied to the chromatographic column. Eluted fractions were collected in 1 mL aliquots, and the enzymatic activity of each fraction was subsequently determined.

### 2.5 Preparation of the Biosensors

Peroxidase from *Cucumis anguria* L. extract was immobilized via covalent bonding onto GA-functionalized TNWs (Figure 1). Aliquots of gherkin homogenates (POX, POX-C, and POX-P), each with a fixed enzymatic loading of 50 U mg^–1^ as determined by prior activity assays, were added to 20 mg of GA-functionalized TNWs. The volume of each enzymatic extract was adjusted based on measured peroxidase activity to ensure consistent enzyme loading across all samples. Normalization was performed using measured enzymatic activity, aiming to achieve comparable functional loading during immobilization rather than biochemical equivalence among the extracts and HRP.

The mixtures were dried for 48 h at room temperature to improve the covalent attachment of the enzyme to the nanowire surface. After immobilization, a step to remove unbound enzymes was performed by washing the TNWs with 1 mL of distilled water and centrifuging for 3 min in Eppendorf tubes. No peroxidase activity appeared in the washing supernatant, indicating minimal enzyme loss during washing and effective enzyme retention on the support within the assay’s detection limits. However, the study did not directly quantify immobilization efficiency or retained enzymatic activity after immobilization, limiting precise evaluation of these parameters. The enzyme-functionalized TNWs were then used to fabricate biosensors. For comparison, commercial HRP was immobilized onto GA-functionalized TNWs using the same procedure.

To prepare the biosensor, graphite powder, mineral oil, and enzyme-functionalized TNWs were combined in a 75:15:10 mass ratio. The components were homogenized in a mortar until a uniform carbon paste was formed. This paste was packed into a carbon paste electrode (CPE) holder with an internal diameter of 3 mm. A control sensor was prepared using TNWs without immobilized peroxidase, following the same procedure. Additionally, biosensors containing TNWs functionalized with commercial HRP were fabricated under identical conditions. All sensors were stored at 12 °C when not in use to maintain enzymatic activity.

### 2.6 Sample Preparation and Detection

A 50 mmol L^–1^ guaiacol stock solution was freshly prepared in PBS (pH 7) before use. DA stock solution (1 mmol L^–1^) was prepared in 0.1 mol L^–1^ PBS (pH 7). Similarly, a 0.0382 mol L^–1^ H_2_O_2_ stock solution was prepared in PBS (pH 7), and 102 μL aliquots were transferred to the electrochemical cell for measurements.

### 2.7 Electrochemical Experiments

Cyclic voltammetry (CV), Square-wave voltammetry (SWV), Electrochemical impedance spectroscopy (EIS) and Differential pulse voltammetry (DPV) were performed using a conventional three-electrode system. The biosensor acted as the working electrode, an Ag/AgCl (3 mol L^–1^ KCl) electrode served as the reference, and a platinum plate (10 mm × 5 mm) was used as the auxiliary electrode. All measurements were carried out at room temperature (25 °C) using an Autolab PGSTAT12 potentiostat/galvanostat (Eco Chemie, The Netherlands). CV analysis of the modified electrode was performed in the presence of 5 mmol L^–1^ Fe­(CN)_6_^3–/4–^ in 0.1 mol L^–1^ KCl solution. This protocol was used for systems utilizing BareCPE, TNW and TNW-POX as working electrodes. SWV measurements: frequency of 100 Hz, amplitude of 100 mV, step of 3 mV. EIS measurements were first conducted over a frequency range of 100 kHz to 0.10 Hz with an amplitude of 10 mV. Following this, the Randles equivalent circuit was used to fit the probe experiment and determine charge transfer resistance (*R*_ct_) associated with the probe’s redox reaction. For DPV measurements, the instrumental parameters were as follows: pulse amplitude, 25 mV; pulse time, 50 ms; and step potential, 5 mV. SWV measurements were conducted in a solution containing 0.2 mmol L^–1^ DA and 2 mmol L^–1^ H_2_O_2_, prepared in 0.1 mol L^–1^ PBS at pH 7.0. Before each measurement, the solution was stirred for 60 s and then allowed to rest for 10 s. A potential of −0.30 V was applied for 3 s before each SWV scan during both calibration curve construction and standard addition experiments (Section 2.8). Repeatability was assessed by conducting five SWV scans with the same biosensor in a 0.1 m L^–1^ DA solution in PBS at pH 7. Reproducibility was assessed using three independently fabricated biosensors, each subjected to five SWV scans under identical experimental conditions. Stability was evaluated by measuring the coefficient of variation on days 1, 15, and 30 after biosensor fabrication, using freshly prepared 100 μmol L^–1^ DA solutions in PBS at pH 7. An interference study was performed using biosensors in the presence of 0.167 mol L^–1^ (10 g L^–1^) urea containing 0.2 mmol L^–1^ DA using SWV, corresponding to the average urea concentration in human urine. Another interference test was assessed by performing the DPV analytical procedure after replacing the 10 μmol L^–1^ DA solution with 10 μmol L^–1^ uric acid (UA). The electroactive area of the electrodes was estimated by CV, based on the relationship between scan rate and current, using the Randles-Ševčík eq 1,^37,38^ as shown in eq 1:

1 $$I_{pa}=2.687\times 10^{5}n^{3/2}AD_{0}^{1/2}C_{0}v^{1/2}$$

Where *I*_pa_ represents the anodic peak current, *n* denotes the number of transferred electrons, *A* is the electroactive area (cm^2^), and *D*_0_ is the diffusion coefficient of ferricyanide in a 5 mmol L^–1^ [K_3_Fe­(CN)_6_] and 0.1 mol L^–1^ KCl solution, valued at 7.03 × 10^–6^ cm^2^ s^–1^.^39^ Additionally, *v* is the scan rate (V s^–1^) and *C*_0_ is the concentration (mol cm^–3^).

### 2.8 Comparative Methods and Dopamine Determination in Pharmaceutical Samples

The concentration of DA in commercial samples was determined by ultraviolet–visible spectrophotometry at 278 nm (λ), following the procedure described in ref 40. A calibration curve was constructed using a 0.3 mmol L^–1^ DA stock solution prepared in distilled water. Aliquots of 100 μL were transferred into cuvettes containing 2.7 mL of distilled water. DA concentrations in pharmaceutical samples were also measured. For the standard addition method, each sample was diluted 10-fold with 0.1 mol L^–1^ PBS (pH 7). A 0.1 mL aliquot of the diluted sample was added to an electrochemical cell containing 2 mmol L^–1^ H_2_O_2_. For quantification, 100, 200, 300, 400, and 500 μL of a standard DA solution (1.5 mmol L^–1^) were sequentially introduced.

DA quantification was conducted using high-performance liquid chromatography (HPLC) on a Shimadzu Prominence system (LC-20AT, CBM-20A). The system was equipped with an SPD-M20A photodiode-array detector, a CTO-20A column oven, and a SIL-10AF autosampler. Chromatographic separation was performed on a Luna Omega column (150 × 4.6 mm, 5 μm, 100 Å, Phenomenex). The mobile phase comprised solvent A (water containing 1% acetic acid) and solvent B (methanol containing 1% acetic acid) in a 9:2 (v/v) ratio. Analyses were performed under isocratic conditions at a flow rate of 1.0 mL min^–1^. The column temperature was 30 °C. The injection volume was 25 μL, and the total run time was 10 min. Detection was achieved using photodiode array acquisition from 200 to 500 nm, with chromatographic quantification at 280 nm (bandwidth 4 nm). The resulting DA peak, eluting at approximately 1.55 min, served as the basis for quantification by peak area, facilitating comparison across calibration and sample analyses. Analytical calibration curves were generated using DA standard solutions in ultrapure water. The concentrations were 4, 8, 12, 16, and 20 ppm. Each was analyzed in duplicate, and the mean peak area was used as the analytical response. This calibration enabled direct interpolation of DA amounts in sample analyses. The pharmaceutical sample was diluted 500-fold with ultrapure water before analysis. DA concentration, determined by interpolating the chromatographic peak area onto the calibration curve, was then adjusted for the dilution factor to yield the final value in mg mL^–1^, ensuring comparability with the calibration standards.

## 3 Results and Discussion

### 3.1 Extracts Characterization

Turning to extraction conditions, the optimal pH for enzymatic activity was determined to be 7; consequently, all gherkin extracts in this study were prepared at this pH. This finding aligns with the literature,^41–44^ which reports optimal extraction pH values for plant-derived peroxidase between 6 and 7. This trend is attributed to the physiological pH of most plant tissues being near 7, a condition that preserves enzyme structure and catalytic efficiency.

The measured enzymatic activities were 8,000 U mL^–1^ for POX, 8,500 U mL^–1^ for POX-C, and 4,500 U mL^–1^ for POX-P. As anticipated, POX-P demonstrated lower enzymatic activity than POX-C, attributable to the peroxidase molecular weight exceeding the ultrafiltration membrane cutoff of 3 kDa. As a result, the enzyme was predominantly retained in the concentrated fraction (POX-C), leading to higher catalytic activity. A 27-day stability study showed that both POX-C and POX-P extracts experienced an approximate 10% decrease in enzymatic activity. Despite this moderate reduction, both fractions maintained substantial catalytic performance, underscoring their robustness and supporting their potential for long-term application in biosensor development as alternative peroxidase sources. In contrast, the commercial HRP solution exhibited lower stability, with enzymatic activity decreasing to approximately 75% of its initial value after 27 days. This reduced stability is likely due to the intrinsic instability of purified enzymes, which, without their native cellular environment and stabilizing biomolecular components, are more prone to structural changes and loss of catalytic function over time.^45^ The superior stability of POX-C aligns with its enhanced biosensor performance, suggesting that partial concentration of the crude extract preserves enzymatic activity and increases robustness.

The molecular mass of gherkin peroxidase (POX) exhibited similar elution profiles on Sephadex G-25 and G-50 columns. On the Sephadex G-75 column, delayed elution suggested interaction with the matrix pores. Instead of a distinct activity peak at 4 mL, activity stabilized up to approximately 5 mL. These findings indicate that POX has a molecular mass between 30 kDa (the upper limit of Sephadex G-50) and 80 kDa (the upper limit of Sephadex G-75). This range is consistent with literature values for plant-derived peroxidases. For example, Pandey et al. (2017)^46^ reviewed 18 studies on plant-derived peroxidases, of which 15 reported molecular masses ranging from 30 to 80 kDa. Collectively, these findings support the estimated molecular mass of POX and align with typical values for plant peroxidases.

While activity assays and gel filtration results indicate the presence of peroxidase-like catalytic species in the extract, these methods do not yield conclusive data on protein composition, enzyme purity, or specific activity. Consequently, the extract is best characterized as a peroxidase-rich plant preparation rather than a biochemically purified enzyme system.

### 3.2 TNWs Characterization

The crystallinity of the synthesized TNWs was confirmed by XRD analysis (Figure S1), a finding further supported by morphological characterization using SEM (Figure 2A). Specifically, diffraction peaks at 2θ values of 10.2°, 25.1°, 37.8°, and 48.1° are indicative of one-dimensional trititanate nanostructures (Na_*x*_H­(2–_*x*_)­Ti_3_O_7_).^34–36^ Notably, the diffraction peak near 2θ = 10° corresponds to the interlayer distance of the lamellar titanate structure, thus confirming the successful formation of titanate nanowires.^35,36^ These results collectively verify the intended one-dimensional structure of the TNWs. Furthermore, SEM images obtained before and after enzyme immobilization showed that the nanowire morphology was preserved, indicating that the immobilization process did not significantly alter the structural organization of the TNWs. This preservation suggests the TNWs are suitable for applications requiring stability during enzyme immobilization.

**Figure 2:**
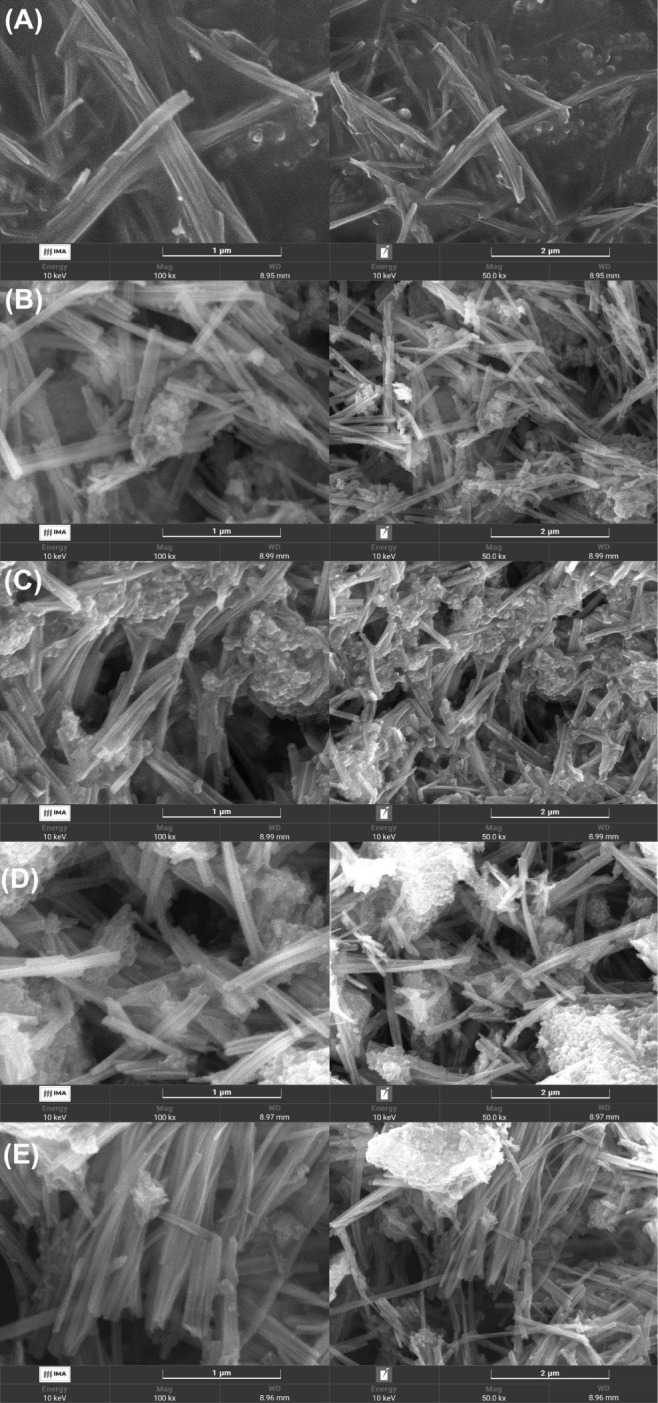
SEM images at magnification 100,000× (left) and 50,000× (right): (A) TNW, (B) TNW-APTMS-GA, (C) TNW-POX, (D) TNW-C, and (E) TNW-P.

SEM/EDX was used to verify the morphology of TNWs, assess surface elemental composition, and monitor changes following peroxidase immobilization. Building on the SEM/EDX results, Figure 2A shows SEM images of TNWs with an average 50 nm diameter. EDX analysis (Figure S2A) confirmed Ti and O as the major elements, with atomic percentages of 10.11% and 39.26%, respectively. The detected oxygen primarily originates from the titanate lattice, with a possible minor contribution from surface hydroxyl groups.^34–36^ These findings match the FTIR spectrum in Figure 3A. Extending the morphological assessment, Figure 2B–E, which correspond to various stages of immobilization with ligands and subsequent incorporation of peroxidases, reveal aggregates adsorbed on the nanowire surfaces, in contrast to the pure TNW shown in Figure 2A. A progressive increase in these aggregates is evident in Figure 2C–E, although the underlying nanowire morphology remains discernible.

**Figure 3:**
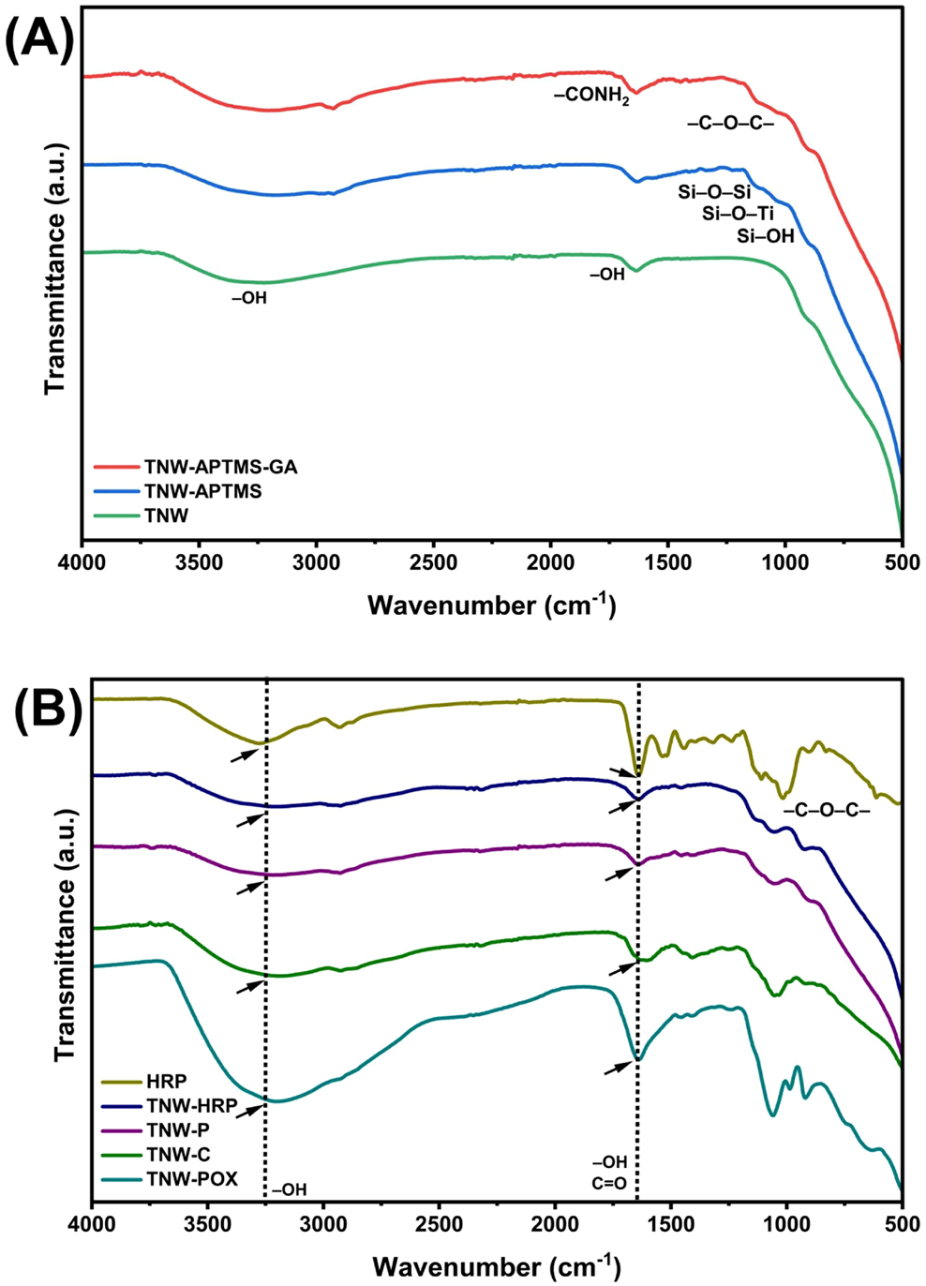
FTIR: (A) TNW without functionalization in green line, TNW functionalized with APTMS (TNW-APTMS) in blue line, and TNW functionalized with APTMS and GA (TNW-APTMS-GA) in red line. (B) TNW functionalized with gherkin (TNW-POX) in dark cyan line, TNW functionalized with gherkin concentrated (TNW-C) in dark green line, TNW functionalized with gherkin filtered (TNW-P) in purple line, TNW functionalized with commercial HRP (TNW-HRP) in navy blue line, and commercial HRP in dark yellow line.

Further exploring the effects of functionalization, Figure 2B depicts TNW functionalized with APTMS and GA ligands. EDX analysis (Figure S2B) indicated that Ti and O remained the principal elements, with atomic percentages of 7.87% and 36.77%, respectively. The observed reduction in Ti percentage relative to pure TNW is likely due to surface coating by adsorbed organic compounds, which complicates EDX quantification. Additionally, N (8.18%) and C (46.45%) were detected, corresponding to the functional groups in APTMS and GA, along with a minor Si signal characteristic of the silanizing agent APTMS. For TNW-POX, EDX (Figure S2C) showed higher O, C, Ti, N, and P at 42.67%, 37.97%, 12.57%, 5.45%, and 1.33%, respectively. The increased O and P are due to the extraction of biomolecules.^30,49^ Trace amounts of Ca, Mg, S, and Na were also present, matching the gherkin extract composition found in TNW-C and TNW-P. In TNW-C (Figure S2D), the main elements were O, C, Ti, N, and P at 44.86%, 34.81%, 11.75%, 7.29%, and 1.33%, respectively. Trace amounts of gherkin extract elements were also detected. Increased O and N show TNW surface uptake of organic compounds,^30,49^ as in the FTIR results (Figure 3B).

Finally, for TNW-P (Figure S2E), the principal elements identified were C, O, N, Ti, and P, with atomic percentages of 53.32%, 32.17%, 7.70%, 6.34%, and 0.47%, respectively. As this sample represents the biosensor after filtration steps, increased retention of organic compounds from the extracts-which are rich in O, N, and P-likely occurred.^30,49^ The relative decrease in Ti content may be attributed to the substantial increase in C, suggesting enhanced organic coating and distinct surface functionalization compared to the other samples.^34,41^

FTIR spectroscopy was conducted to confirm the formation of covalent bonds between TNWs and peroxidases (TNW-POX, TNW-P, TNW-C, and TNW-HRP). Figures 3A and B show the FTIR spectra for each stage of TNW functionalization. In the first step, APTMS was used to introduce −NH_2_ groups onto the TNW surface (TNW-APTMS). In the second step, GA was employed to attach −CHO groups (TNW-APTMS-GA), which subsequently reacted with the −NH_2_ groups of peroxidase, forming a covalent bond between the enzyme and the nanowires.^34^ The FTIR results for the TNWs aligned with previous literature and confirmed the successful functionalization and enzymatic immobilization of peroxidase on the TNWs. The spectrum of the original TNWs shows a broad band around 3000–3500 cm^–1^, attributed to −OH groups on the TNWs surface, and a band at 1630 cm^–1^, attributed to the angular deformation of water molecules (Figure 3A).

The first functionalization step with APTMS adds the Si–O–Ti, Si–O–Si, and Si–OH groups to the TNW surface at 1030, 1120, and 907 cm^–1^, respectively, along with several bands between 1200 and 1600 cm^–1^. After the GA step, these smaller bands disappear, but there is an increase at 1640 cm^–1^, attributed to the −CONH_2_ groups, and at 1060 cm^–1^ related to primary alcohol (−OH) stretching or −C–O–C– bond stretching.

A broad absorption band in the 3000–3500 cm^–1^ region is assigned to stretching vibrations of O–H and N–H groups (Figure 3B). These groups are linked to adsorbed water molecules and protein functional groups. The band near 1641 cm^–1^ corresponds to the amide I vibration, which is primarily associated with the C=O stretching of peptide bonds in proteins.^47,48^ The increased intensity of this band in TNW-based materials confirms the presence of immobilized protein structures on the TNWs surface. Additional bands in the 1200–1000 cm^–1^ region, particularly at 1060 cm^–1^ and 1016 cm^–1^, are attributed to C–O–C and C–O stretching vibrations.^34^ These likely originate from alcohol groups, carbohydrate residues, or other oxygenated functional groups present in proteins or plant biomolecules.^34,41^

Comparison of TNW-C and TNW-P reveals distinct spectral differences: TNW-C exhibits more intense absorption bands, particularly in regions corresponding to protein functional groups, such as the amide band and hydroxyl vibrations (Figure 3B). This observation suggests a higher concentration of proteinaceous material in this fraction. This outcome aligns with the membrane filtration process used during extract preparation (TNW-P), in which proteins with molecular masses above the membrane cutoff are preferentially retained. Consequently, a significant portion of peroxidase molecules remains in the retained fraction (TNW-C), leading to stronger FTIR signals from protein functional groups.

When TNW-P is compared with TNW-HRP, the spectra exhibit similar absorption profiles, especially in the regions associated with amide and hydroxyl vibrations.^47,48^ This similarity indicates that the biomolecules immobilized from the crude extract possess structural features comparable to those of purified HRP. However, slight differences in band shape and intensity are observed in TNW-C. These variations can be attributed to the presence of additional biomolecules in the crude plant extract, since extracts obtained from gherkin (*Cucumis anguria* L.) may contain other proteins, enzymes, carbohydrates, and phenolic compounds besides peroxidase.^30,49^ These components may give rise to additional FTIR bands or alter the relative intensities of protein-related signals.

Stronger protein bands in TNW-based materials suggest significant interactions between enzyme groups and the TNW surface, likely via hydrogen bonding or covalent attachment.^34,41^ These interactions improve biosensor performance by enhancing enzyme retention, stability, and sensor response. FTIR provides spectroscopic evidence for enzyme immobilization on the TNW surface, shown by enhanced amide bands. However, FTIR alone is not definitive, as some spectral changes may result from nonspecific TNW interactions. The pronounced spectral features of TNW-HRP indicate that the covalent immobilization strategy^50^ enables effective enzyme attachment and stabilization. These findings show that nanowire biofunctionalization is effective and suitable for the development of the proposed electrochemical biosensor.

Table 1 presents the zeta potential values of the nanomaterials before and after each functionalization step. TNWs display a negative surface charge (−44.7 ± 0.8 mV), attributed to surface hydroxyl groups (−OH), which can donate protons and create a negatively charged surface. Functionalization with APTMS replaces some of these hydroxyl groups with amine groups (−NH_2_), resulting in an inversion to a positive surface charge (+4.3 ± 0.8 mV), as the amine groups can accept protons, creating a positive surface. This behavior is consistent with the literature.^35^ Next, modification with GA alters the surface charge, which becomes negative again (−17.8 ± 0.9 mV) due to the introduction of aldehyde groups (−CHO).

**Table 1:** Zeta Potential (mV) Values for TNW after the Functionalization Steps

Step	TNW	TNW-ATPMS	TNW-APTMS-GA	TNW-P	TNW-C	TNW-HRP
**Zeta potential (mV)**	–44.7 ± 0.8	+4.3 ± 0.8	–17.8 ± 0.9	–13.1 ± 0.7	–22.2 ± 1.0	–19.8 ± 0.5

Following this, immobilization of the extracts and the commercial enzyme, along with subsequent washing steps, results in zeta potential values of −13.1 ± 0.7 mV (TNW-P), −22.2 ± 1.0 mV (TNW-C), and −19.8 ± 0.5 mV (TNW-HRP). The HRP enzyme possesses an isoelectric point at pH 7.2 ^40^ and is therefore predominantly negatively charged under the experimental conditions (pH 7). This attribute aligns with the observed value for TNW-HRP, which exhibits a negative zeta potential (−19.8 mV), slightly lower than that of the functionalized material without enzyme (TNW-APTMS-GA, −17.8 ± 0.9 mV), extending the trend noted in the previous step. Nanomaterials containing plant extracts showed variations in zeta potential. TNW-P exhibited a reduction in the magnitude of the negative charge (−13.1 ± 0.7 mV), indicating that components in the extract may decrease the net negative surface charge. In contrast, TNW-C displayed an increase in negativity (−22.2 ± 1.0 mV), suggesting that some extract components enhance the negative charge. Given that equivalent amounts of peroxidase activity were used in both cases, these differences are likely attributable to other extract constituents, which may have various charges or influence the amount of protein adsorbed.

The FTIR spectra (Figure 3B) indicate increasing absorption intensity in the order TNW-P < TNW-HRP < TNW-C. The zeta potential data and electrophoresis results support these findings. Together, they suggest that the immobilized proteins have a net negative charge under the experimental conditions. The greater amount of protein adsorbed in the TNW-C material accounts for its more negative zeta potential value compared to TNW-P.

### 3.3 Electrochemical Characterization of the Biosensor

Figure 4A–D displays the cyclic voltammograms for the electrodes at a scan rate of 150 mV s^–1^. The TNW-POX biosensor exhibited higher anodic (*I*_pa_) and cathodic (*I*_pc_) peak currents compared to the other electrodes. The incorporation of TNWs resulted in an increase in peak currents by approximately 57% for *I*_pa_ and 60% for *I*_pc_, relative to the bareCPE (*I*_pa_: 47.1 μA to 73.8 μA; *I*_pc_: −54.8 μA to −87.4 μA), which demonstrates enhanced electron-transfer properties of the nanomaterial. Subsequent enzyme immobilization led to a further increase in peak currents by approximately 11% for *I*_pa_ (82.3 μA) and 9% for *I*_pc_ (−94.9 μA), indicating an additional catalytic effect attributable to the immobilized enzyme.

**Figure 4:**
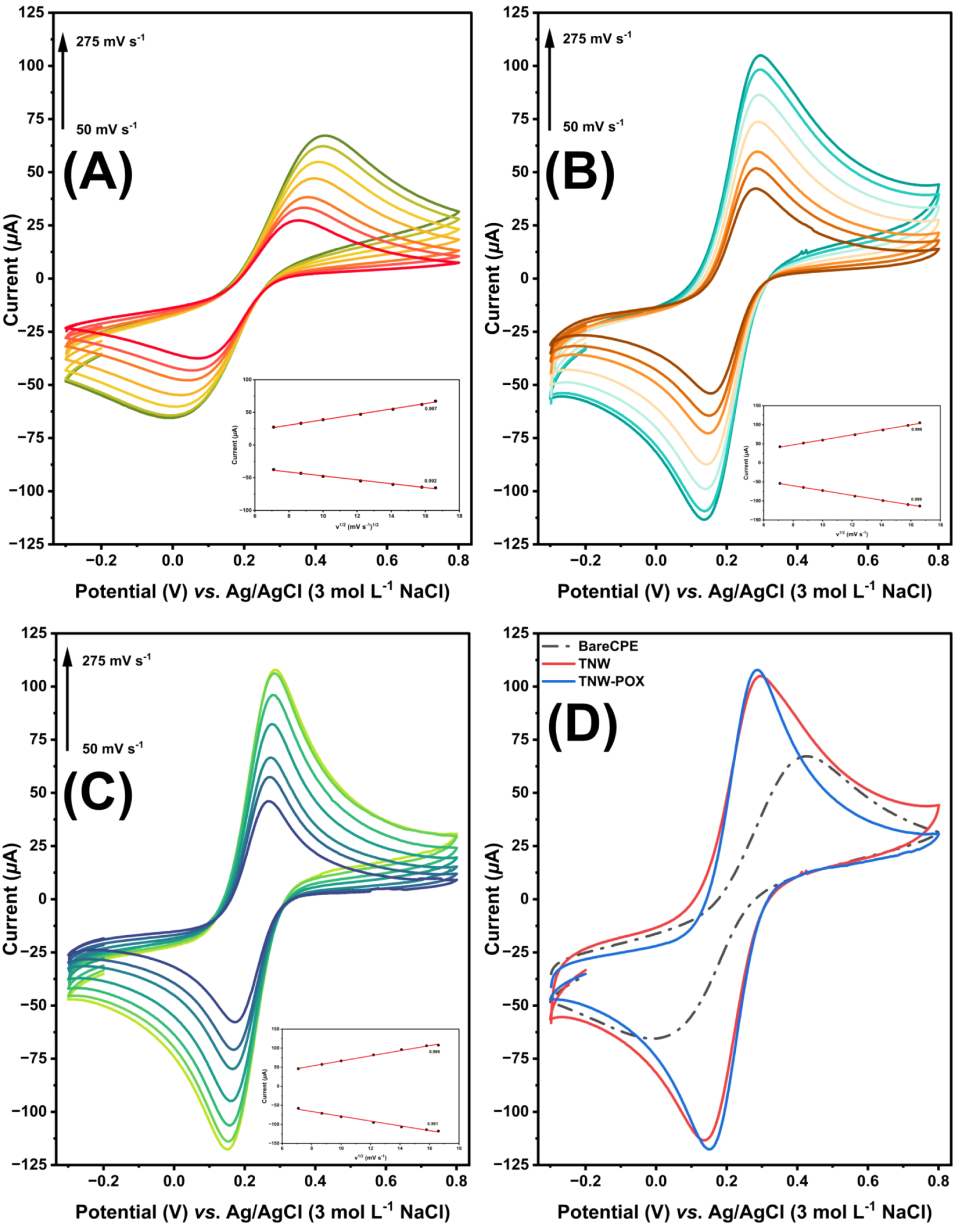
Cyclic voltammograms of the electrodes in a solution of 5 mmol L^–1^ K_3_Fe­(CN)_6_ in 0.1 mol L^–1^ KCl at scan rates from 25 to 400 mV s^–1^: (A) BareCPE, (B) TNW, (C) TNW-POX, and (D) Comparison (150 mV s^–1^).

A comparable increase in current was seen when commercially available HRP was immobilized under identical conditions. This suggests the behavior is typical of peroxidase-based systems rather than unique to the plant extract. Previous studies using TNW-based electrodes with HRP have also reported enhanced electrochemical responses compared to nonenzymatic systems, confirming the catalytic role of immobilized peroxidases.^34–36^ Furthermore, this interpretation is strengthened by recent modeling studies of HRP-based biosensors, which demonstrate that immobilized peroxidase actively contributes to the measured current via catalytic mechanisms.^23^ Collectively, both the experimental findings and the literature evidence indicate that the observed signal enhancement is, at least in part, mediated by the enzyme.

Regarding of peak separation (Δ*E*_p_), the TNW-POX biosensor resulted in a value of 115 mV for the Fe­(CN)_6_^3–/4–^ redox pair, while TNW and BareCPE displayed Δ*E*_p_ values of 146 and 371 mV, respectively. These results suggest that incorporating TNWs and the immobilized biocatalytic extract improves the electrochemical response of the electrode, as indicated by higher peak currents and lower Δ*E*_p_ values relative to the unmodified CPE, likely due to an increase in effective electroactive area and a more favorable interfacial charge-transfer environment. The linear relationship between *I*_pc_ (*R* ^2^ = 0.996) and *I*_pa_ (*R* ^2^ = 0.998) and the square root of the scan rate indicates a diffusion-controlled electrochemical process in accordance with the Randles-Ševčík equation. The electrochemical area was estimated to be 0.0375 cm^2^ for the BareCPE electrode, 0.0557 cm^2^ for the TNW electrode, and 0.0570 cm^2^ for the TNW-POX biosensor. These results indicate that TNWs significantly increase the electrode’s effective area.

Although mass transport can occur through diffusion, migration, and convection, experimental conditions in voltammetry measurements are controlled to ensure that only diffusion is present. Convection is eliminated because the solution remains stationary during scanning.^51^ Migration, defined as the movement of ions driven by electrostatic interactions between solution species and the working electrode, is minimized by the presence of supporting electrolytes in the buffer solution, which are at a concentration significantly higher than that of the analyte. Therefore, the observed result (as shown in Figure 4C) that the biosensor operates under diffusion-controlled conditions, is consistent with theoretical expectations.^52^

Surface modifications in the biosensors were evaluated by measuring *R*_ct_ using EIS for each developed system (Figure 5A). The BareCPE electrode showed an initial *R*_ct_ value of 585 Ω cm^2^, while the TNW electrode exhibited a slightly higher *R*_ct_ of 611 Ω cm^2^. Although TNW increases surface area, facilitates extract immobilization, and demonstrates biocompatibility, its semiconductor properties and moderate conductivity result in a similar *R*_ct_ to that of BareCPE. This means that adding TNWs alone did not significantly reduce *R*_ct_ relative to BareCPE, despite the nanostructure providing an improved voltammetric response due to a greater electroactive area and enhanced interfacial characteristics.^34–36^

**Figure 5:**
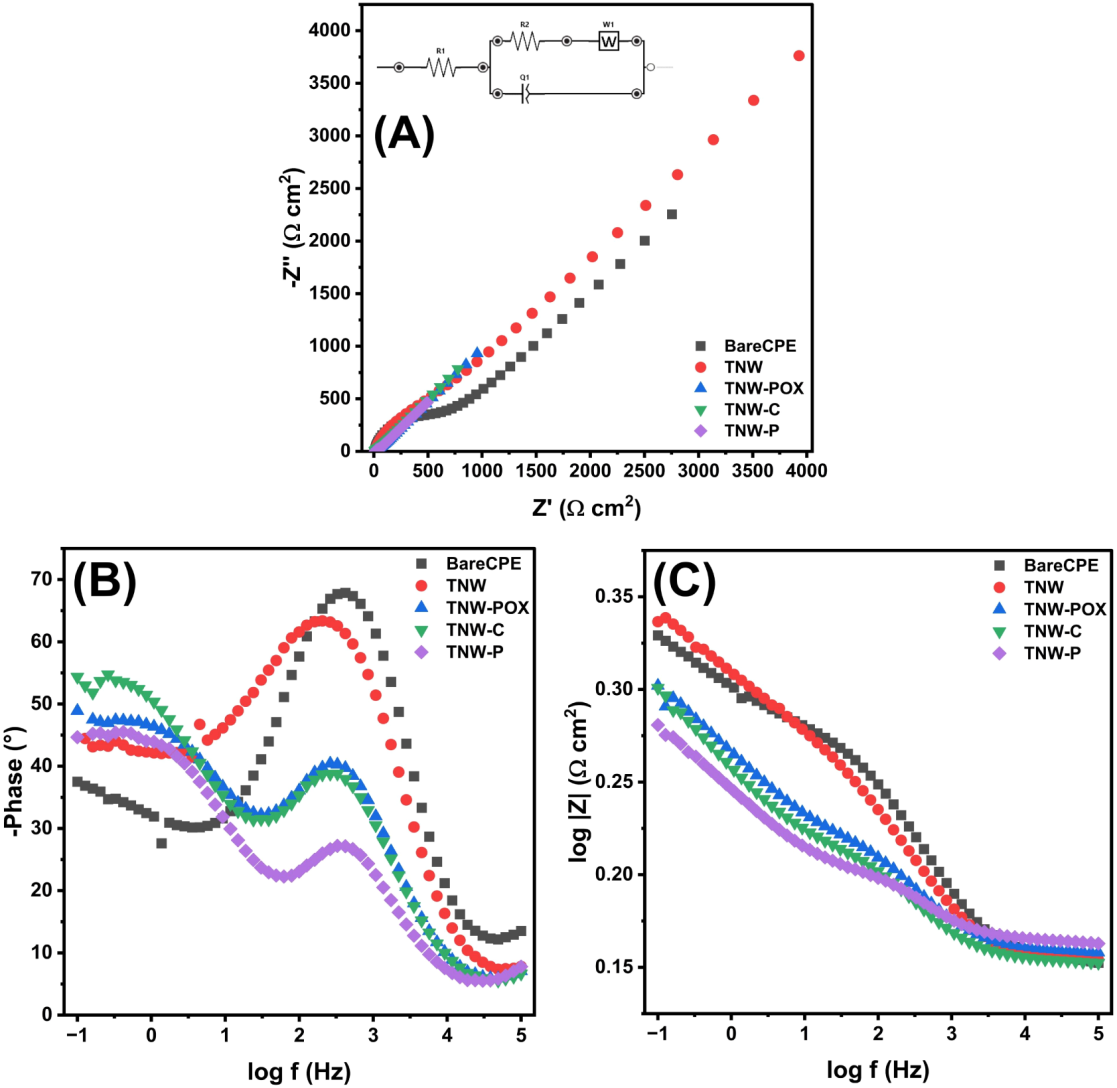
EIS spectra. (A) Nyquist plot, (B) and (C) Bode plot recorded at different modification stages in a solution of 5:5 mmol L^–1^ K_3_Fe­(CN)_6_ in 0.1 mol L^–1^ KCl. Adjust: Randle’s equivalent circuit with Q phase.

Following extract immobilization, *R*_ct_ values decreased markedly: TNW-POX (54 Ω cm^2^), TNW-C (49 Ω cm^2^), TNW-P (24 Ω cm^2^). This pronounced decrease demonstrates successful peroxidase immobilization and enhanced electron conduction, providing clear evidence of improved charge-transfer efficiency at the electrode/electrolyte interface.^34,41^ The TNW-P biosensor exhibited the lowest *R*_ct_ value among all tested configurations, demonstrating the most enhanced electron-transfer efficiency in direct comparison. This improvement is likely attributable to lower-molecular-weight redox compounds, increased availability of the enzymatic fraction, improved extract dispersion, and the formation of a more uniform film on the electrode surface.^30,49^ Conversely, compared to TNW-P, the TNW-C biosensor may contain higher proportions of fibers, polysaccharides, high-molecular-weight proteins, and colloidal material, which could partially block the surface, increase the diffusion barrier, and hinder electron transfer.^30,49^ The observed *R*_ct_ values align with previous expectations, indicating the electrocatalytic activation effect of the gherkin extract and demonstrating improved charge transfer after biofunctionalization. The reduction in *R*_ct_ after modification with gherkin extract, when compared to electrodes without extract, suggests that the bioactive components in the extract favor electron-transfer kinetics at the electrode/electrolyte interface.

Consistent with the Nyquist diagram (Figure 5A), the Bode diagrams (Figure 5B–C) showed significant changes in −Phase angle (°) after electrode surface modification. The BareCPE electrode had a phase angle of 68°, indicating mostly capacitive behavior, as this approaches 90°, typical for such interfaces. Following TNW incorporation, the Nyquist diagram showed a high *R*_ct_, while the Bode diagram showed an increase in the −Phase angle (°) to 63°. This shift suggests the onset of more resistive behavior, likely associated with interfacial modifications and partial facilitation of electron transfer. In comparison to both BareCPE and TNW electrodes, incorporating bioactive extracts resulted in further increases in −Phase angle (°): TNW-POX (40°), TNW-C (39°), and TNW-P (27°), as seen in the Nyquist diagram. These progressive increases contrast the 68° of BareCPE and 63° of TNW, indicating reduced capacitance and enhanced electron transfer. These differences also provide evidence of biomolecule immobilization, biological film formation, and changes in double-layer capacitance.^30,31^ The frequency (Hz) at which phase peaks occurred also shifted in a clear sequence (Figure 5B). While the TNW electrode’s phase peak occurred at 206.1 Hz, bioactive extract incorporation increased peak frequencies to TNW-POX (262.4 Hz), TNW-C (262.4 Hz), and TNW-P (328.2 Hz). Compared to the lower frequency of TNW, this sequential increase demonstrates progressively enhanced electron-transfer kinetics and electrocatalytic activity, matching the trend of decreasing *R*_ct_ in the Nyquist diagram. Changes in −Phase angle (°) peaks, including profile modifications and broadening, were attributed to surface roughness, biofunctionalization, and interactions between extract biomolecules and the nanostructured electrode.

### 3.4 Influence of Experimental Parameters on Biosensor Performance

The influence of experimental parameters on the performance of four proposed biosensors was investigated by systematically evaluating key variables, including pH (6–8), SWV settings such as frequency (40–100 Hz), and pulse amplitude (40–100 mV). The highest current response was achieved using a 0.1 mol L^–1^ phosphate buffer solution at pH 7 (86.4 ± 0.3 μA), containing 0.2 mmolL^–1^ DA and 2 mmol L^–1^ H_2_O_2_, a frequency of 100 Hz (34.2 ± 0.4 μA), and a pulse amplitude of 100 mV (57.7 ± 0.2 μA). These conditions were employed for all subsequent measurements (Figure S3A–C). Variations in the optimal pH for peroxidase activity on TNW are attributable to differences in enzyme binding modes and the unique surface characteristics of each nanomaterial. This behavior may also reflect microenvironmental effects created by the nanostructured support, which can alter the apparent optimal pH of immobilized enzymes. In systems employing covalent immobilization on TNWs, the enhanced stability observed at neutral pH likely results from the strong anchoring of the enzyme to the support. Consequently, differences in binding strategy and surface composition explain the distinct pH preferences observed for enzymatic activity.

### 3.5 Analytical Performances

Calibration curves for DA were generated in triplicate using the proposed biosensor under optimized SWV conditions (Figure S3A–C). The analytical methodology was validated in accordance with the International Council for Harmonization (ICH) guidelines.^53^ Sensitivity was determined by dividing the slope of the calibration curve (*S*) by the electrode’s active geometric area (*A*_geo_), according to the relationship: Sensitivity = *S*/*A*_geo_.^54^

The resulting plots (Figure 6A–H) demonstrated a linear response for the TNW-POX biosensor over the concentration range of 5 to 65.4 μmol L^–1^, described by the equation *I*_pa_ = 1.14_DA_ – 4.47 (*R* ^2^ = 0.997). The limit of detection (LOD) was 1.44 μmol L^–1^, the limit of quantification (LOQ) was 4.36 μmol L^–1^ and sensitivity (*S*) was 16.32 μA μmol L^–1^ cm^–2^. For the TNW-P (5–56.6 μmol L^–1^), TNW-C (5–52.1 μmol L^–1^), and TNW-HRP (5–56.6 μmol L^–1^) biosensors, the following analytical parameters were obtained, respectively: *I*_pa_ = 0.744_DA_ + 2.35 (*R* ^2^ = 0.994), with a LOD 2.20 μmol L^–1^, LOQ of 6.66 μmol L^–1^ and a *S* 10.63 μA μmol L^–1^ cm^–2^; *I*_pa_ = 0.573_DA_ + 2.44 (*R* ^2^ = 0.999), with a LOD of 0.400 μmol L^–1^, a LOQ of 1.21 μmol L^–1^, and a *S* of 8.19 μA μmol L^–1^ cm^–2^; *I*_pa_ = 0.571_DA_ + 0.497 (*R* ^2^ = 0.998), with a LOD of 0.694 μmol L^–1^, a LOQ of 2.10 μmol L^–1^, and a *S* of 8.15 μA μmol L^–1^ cm^–2^. The linear range starts above the lowest physiological dopamine concentrations, but the detection limit remains within the biologically relevant range, especially for urinary levels, supporting its use in analytical applications. While SWV offers high sensitivity and rapid analysis, future studies should incorporate DPV measurements to further enhance the assessment of sensor performance by providing improved resolution between analyte signals.

**Figure 6:**
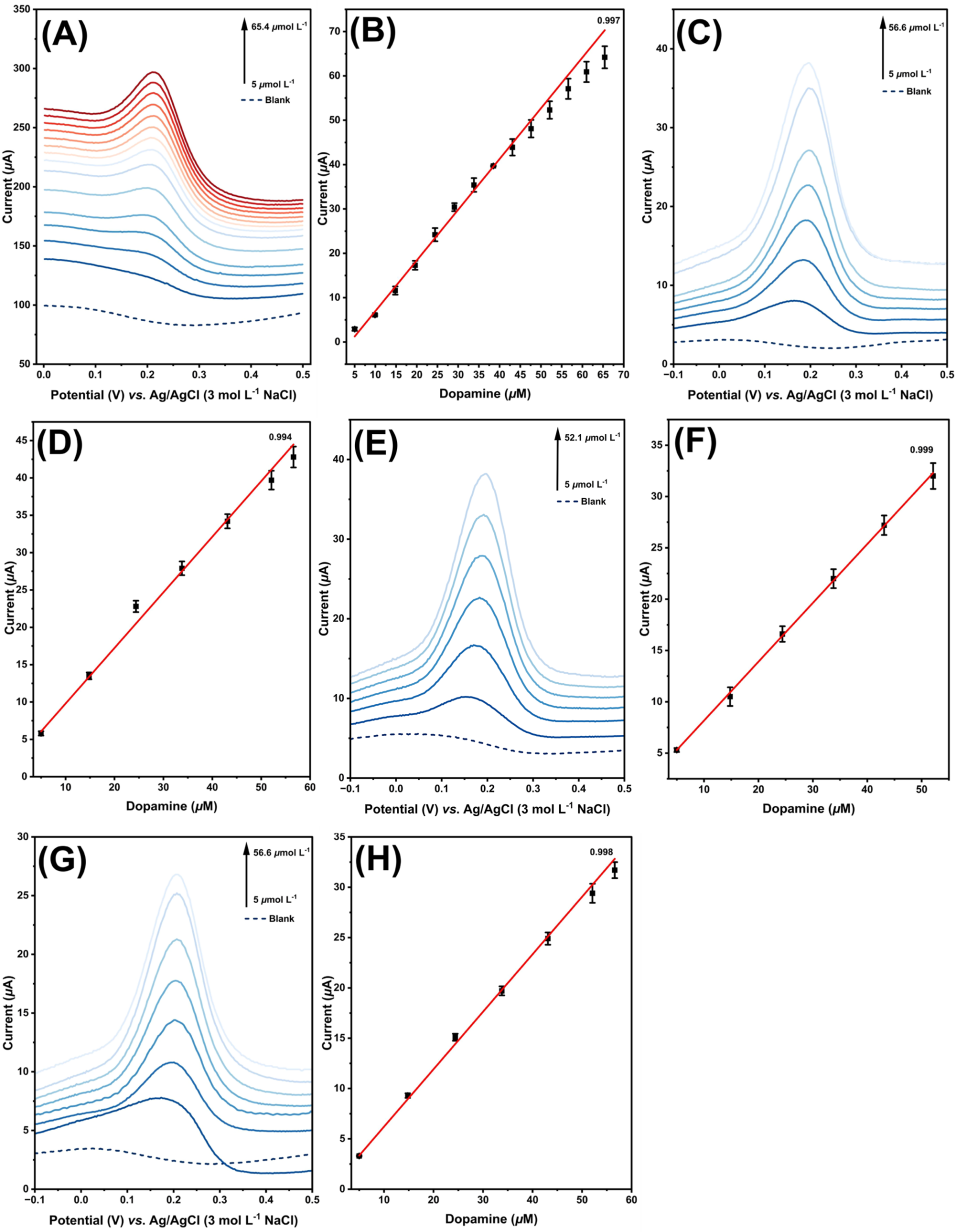
Calibration plot for DA in H_2_O_2_ 2 mmol L^–1^ and 0.1 M PBS pH 7. Parameters of SWV: frequency of 100 Hz, amplitude of 100 mV, step of 3 mV. (A) and (B) TNW-POX biosensor, (C) and (D) TNW-P biosensor, (E) and (F) TNW-C biosensor, (G) and (H) TNW-HRP biosensor (*n* = 3).

Beyond the analytical parameters derived from calibration curves, distinct differences in voltammetric peak profiles were observed among the biosensors (Figures 6A, C, E and G). The TNW-POX electrode displayed a broader, less defined half-height width compared with the other systems (TNW-C, TNW-P, and TNW-HRP), which exhibited comparable peak shapes. In SWV, the half-peak width reflects the kinetics of electron transfer and the uniformity of the electroactive surface. Broader peaks typically indicate a less homogeneous electrochemical environment or slower electron transfer kinetics.^55^

Building on these observations, the wider peak observed for TNW-POX can be attributed to the more complex composition of the crude enzymatic extract immobilized on the TNW surface. Plant extracts obtained from gherkin (*Cucumis anguria* L.) can contain various biomolecules besides peroxidase, including other enzymes, proteins, polysaccharides, and phenolic compounds.^30^ The presence of these additional components can lead to a more heterogeneous biofilm on the electrode surface, potentially partially hindering electron transfer and broadening the voltammetric peak.^55^ Although TNW-POX showed the highest sensitivity (16.32 μA μmol L^–1^ cm^–2^), it had a relatively higher LOD (1.44 μmol L^–1^) compared to TNW-C and TNW-HRP. The higher sensitivity suggests that the presence of multiple biomolecules in the crude extract may increase the overall catalytic activity or the number of electroactive sites available for DA oxidation. However, the broader peak profile and greater surface heterogeneity may increase background variation, potentially lowering the limit of detection.

In contrast to TNW-POX, TNW-C and TNW-HRP produced narrower, well-defined peaks, reflecting a more homogeneous electrochemical interface and uniform electron-transfer processes.^56^ This characteristic may account for the lower detection limits observed for these biosensors, especially for TNW-C, which achieved the lowest LOD (0.400 μmol L^–1^). A more defined peak profile enhances signal resolution and minimizes noise, thereby supporting lower detection limits even when overall sensitivity is marginally reduced.

All immobilization experiments used the same initial enzymatic loading (50 U mg^–1^ of GA-functionalized TNWs) to ensure a controlled comparison among POX, POX-C, POX-P, and HRP biosensors. However, because we did not directly quantify immobilized activity or activity recovery, we cannot determine if observed performance differences among biosensors result from intrinsic differences in extract composition or stability, or from differences in immobilization effectiveness.

Overall, these findings show that TNW-POX benefits from multiple biomolecules, boosting catalytic activity, while more homogeneous enzyme immobilization in TNW-C and TNW-HRP produces sharper voltammetric responses and better detection limits. Thus, enzyme purity and surface organization significantly affect biosensor performance. The detection limits ranked TNW-C < TNW-HRP < TNW-POX < TNW-P. TNW-C, using concentrated gherkin extract, slightly outperformed the others, even surpassing TNW-HRP and matching the performance of purified commercial enzymes when immobilized on a nanostructured support.

Based on literature reports, a plausible sensing mechanism involves the synergistic interaction between enzymatic catalysis and electrochemical transduction (Figure 7). DA undergoes oxidation to DA-*o*-quinone via a two-electron, two-proton process, which proceeds through two sequential one-electron steps involving a semiquinone intermediate.^57^ Plant-derived peroxidase, which contains a heme iron center in the Fe^3+^ state, is activated by H_2_O_2_ in solution to generate high-valent intermediates, specifically Compound I and Compound II (Fe^4+^=O, ferryl species).^58,59^ These intermediates serve as potent oxidizing agents, promoting the stepwise oxidation of DA. During this process, electrons released from DA are transferred to the electrode surface via the conductive network of TNWs, thereby enhancing electron transport and improving signal transduction.^60^ The enzyme is then regenerated to its native Fe^3+^ state, thereby completing the catalytic cycle. This integrated mechanism produces an amplified electrochemical response, allowing for sensitive detection of DA.

**Figure 7:**
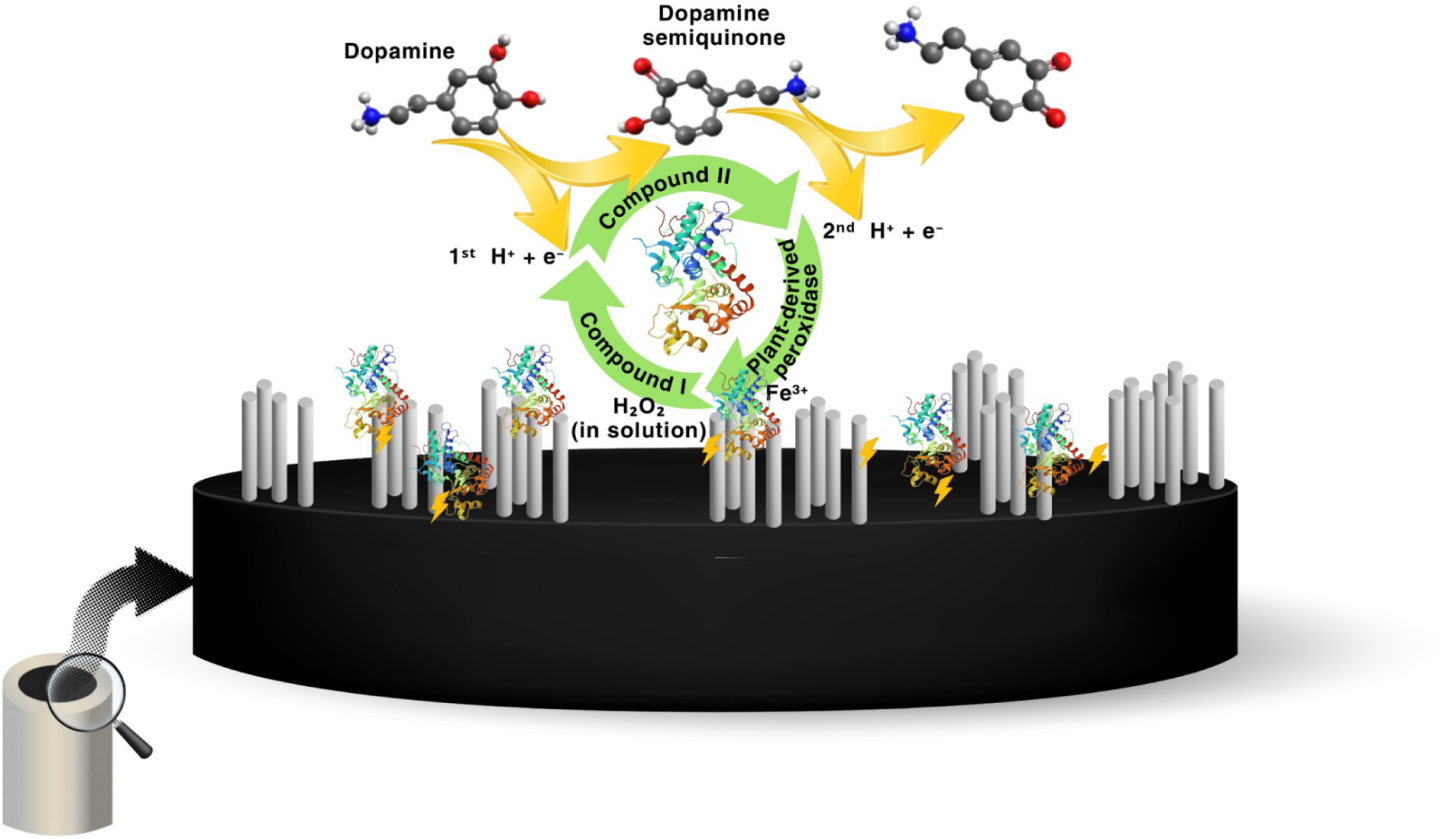
A schematic shows DA oxidized to DA-*o*-quinone via a two-electron, two-proton process catalyzed by immobilized plant-derived peroxidase on TNWs, which transfer electrons to the electrode, enabling highly sensitive detection of DA.

### 3.6 Reproducibility, Repeatability, and Stability

The TNW-POX biosensor demonstrated high repeatability, yielding a mean peak current of 101.3 ± 1.5 μA with a relative standard deviation (RSD) of 1.5%. In comparison, TNW-P, TNW-C, and TNW-HRP biosensors produced mean peak currents of 32.4 ± 0.7 μA (RSD = 2.2%), 32.7 ± 0.8 μA (RSD = 2.4%), and 33.7 ± 1.3 μA (RSD = 3.8%), respectively. Reproducibility was evaluated using independently fabricated biosensors. The TNW-POX biosensor achieved a mean peak current of 104 ± 4 μA and an RSD of 4%, indicating good fabrication consistency. The TNW-P, TNW-C, and TNW-HRP biosensors exhibited mean peak currents of 32.4 ± 1.6 μA (RSD = 5%), 31.2 ± 4.3 μA (RSD = 2.4%), and 35.6 ± 3 μA (RSD = 8.4%), respectively.

The TNW-POX biosensor showed a 17% decrease in peak current after 15 days and retained 79% of its initial signal after 30 days. Similar findings have been reported,^34^ where a biosensor based on commercial peroxidase immobilized on TNW for H_2_O_2_ detection retained approximately 91% of its original signal after 30 days.

Using the same stability evaluation protocol applied to the TNW-POX biosensor, the TNW-P, TNW-C, and TNW-HRP biosensors were tested with freshly prepared 50 μmol L^–1^ DA solutions at pH 7. With an initial signal of approximately 40 μA on day one, the TNW-P and TNW-C biosensors retained 58% of their initial signal after 30 days, while the TNW-HRP biosensor maintained 50%. In a separate study,^61^ biosensors used continuously over 30 days retained less than 65% of their initial response, whereas devices used only on the 30th day preserved about 10% of the original signal. These findings support the hypothesis that the presence of native biomolecules in crude or semipurified extracts acts as a protective microenvironment, enhancing enzyme stability and long-term biosensor performance.

### 3.7 Interference Test

The presence of urea (10 g L^–1^) caused relative signal changes of −6%, −0.9%, and −5% for TNW-P, TNW-C, and TNW-HRP, respectively. The slight decrease in signal observed in the presence of urea reflects only a modest, nonspecific effect, most likely due to urea’s chaotropic properties^62^ and not a meaningful inhibition of the immobilized enzyme (weak inhibitor). Urea was selected as an interfering compound due to its high concentration and relevance in urine analysis.^63^

Among the devices tested, the TNW-C biosensor demonstrated significantly superior performance, whereas TNW-P and TNW-HRP exhibited similar responses. This result is likely due to the increased stability of POX in the TNW-C system, which retains most extract components in a concentrated form during preparation. Previous studies have shown that enzymes often display greater stability when present in crude extracts, due to the presence of protective proteins and other biomolecules that preserve enzymatic structure and enhance catalytic efficiency.^42,45^

Due to their minimal and maximal interference with urea (−0.9% for TNW-C, −6% for TNW-P), TNW-C and TNW-P were chosen for further selectivity testing via DPV in the presence of UA, a common interferent for DA detection. Using equimolar dopamine and UA (10 μmol L^–1^, 1:1), both produced well-resolved oxidation peaks (Figure S4). Oxidation potentials were +0.16 V for DA and +0.30 V (TNW-C) or +0.29 V (TNW-P) for UA (vs Ag/AgCl). For TNW-C, the DA oxidation current (*I*_pa_ = 0.364 μA) was 3.68 times higher than for UA (*I*_pa_ = 0.099 μA); TNW-P’s DA current (0.320 μA) was 2.18 times higher than for UA (*I*_pa_ = 0.147 μA). The well-separated oxidation peaks and higher DA current indicate good biosensor selectivity, consistent with the urea interference study and overall analytical performance. Extract content differences between TNW-C and TNW-P, due to concentration and filtration, may influence these electrochemical responses.

### 3.8 Comparative Performance Analysis of DA (Bio)­sensors

Table 2 compares the biosensors developed in this study with previously reported sensors and biosensors for DA determination.

**Table 2:** Comparison of (Bio)­sensors for DA Determination

Electrode	Method	Linear range^a^	Detection limit^a^	Sensitivity^b^	Ref
GR45CO15	CV^c^	0.37–100	0.399	1.295	^2^
	DPV^d^	0.94–18.5	0.938	1.725	
CPE/Banana tissue/MWCNT	DPV	10–30	2.09	–	^4^
MWCNTs/ *Cucurbita pepo* L.	SWV^e^	32–44	2	16.52	^43^
Graphene	DPV	4–100	2.64	0.065	^64^
Au nanospikes	DPV	1–100	5	–	^65^
AgNP–rGO/GCE	LSV	10–1000	5.4	5.57	^66^
sG/GCE	DPV	20–400	2.8	0.165	^67^
CPE/Catalase/ZnO	CV	5–31	3	–	^68^
PLA-G_NaOH‑30‑EC_	CV	10–500	3.49	0.60	^69^
	DPV	7–100	2.57	1.07	
	SWV	5–100	1.67	11.28	
UOx/MWCNT-CMC/Au	CV	20–2700	2.8	0.233	^70^
PANI/1Tm:ZnO	CV	0.8–6.5	1.92	0.2568	^71^
CuNi-MOF@rGO	AMP^f^	1–500	9.42	0.019	^72^
TNW-POX	SWV	5–65.4	1.44	16.32	This work
TNW-P		5–56.6	2.20	10.63	
TNW-C		5–52.1	0.400	8.19	
TNW-HRP		5–56.6	0.694	8.15	

a μmol L^–1^.

b μA μmol L^–1^ cm^–2^.

c Cyclic voltammetry.

d Differential pulse voltammetry.

e Square wave voltammetry.

f Amperometry. CPE: carbon paste electrode. GCE: glassy carbon electrode. MWCNTs: multiwalled carbon nanotubes. sG: solution-exfoliated graphene. G: graphene. PLA: polylactic acid. UOx: urate oxidase. CMC: carboxymethyl cellulose. Au: gold. PANI: polyaniline. 1Tm:ZnO: zinc oxide doped with thulium. MOF: metal–organic framework. GR: graphite. CO: castor oil. TNW: titanate nanowires. POX: gherkin extract (*Cucumis anguria* L.). TNW-P: filtered. TNW-C: concentrated. HRP: horseradish peroxidase.

The four biosensors presented here exhibited satisfactory analytical performance, with detection limits and linear response ranges comparable to those reported for other electrochemical sensors in the literature. The enhanced response observed for the proposed systems can be attributed to the catalytic activity of peroxidase in the presence of H_2_O_2_ combined with the high surface area and favorable electron-transfer properties of titanate nanowires, which facilitate charge transport at the electrode interface.

### 3.9 Commercial Samples

Commercial samples were analyzed using the standard-addition method under previously optimized SWV conditions. An analytical calibration curve was constructed using standard DA solutions over the concentration range of 10.7 to 68.6 μmol L^–1^ for UV–vis spectrophotometry and 26.1 to 130.6 μmol L^–1^ (4–20 ppm) for HPLC. The resulting linear equation was ABS = 0.0024_DA_ + 0.0019 (*R* ^2^ = 0.999) and Area = 17742_DA_ – 10508 (*R* ^2^ = 0.999). Each point of the UV–vis calibration curve represents the mean of three independent measurements (Figure S5A) and each point on the HPLC curve corresponds to the average of two injections (Figure S5B). The DA concentration in the commercial sample for UV–vis spectrophotometry was 4.97 ± 0.04 mg mL^–1^ (3.25 × 10^4^ ± 0.03 μmol L^–1^), determined at a 95% confidence level, which closely matches the nominal concentration of 5 mg mL^–1^ (3.26 × 10^4^ μmol L^–1^) specified by the manufacturer. HPLC was 5.59 ± 0.0002 mg mL^–1^ (3.65 × 10^4^ ± 0.0001 μmol L^–1^), corresponding to a relative error of 11.8% in relation to the labeled value. Table 3 summarizes the results for DA determination in the commercial ampule using both the prepared biosensor, the spectrophotometric, and the chromatography method. The equations obtained from the standard addition of DA to the commercial sample, as shown in Figure S6A–D, were *I*_pa_ = 1.08_DA_ + 28.5 (*R* ^2^ = 0.997) for TNW-POX, *I*_pa_ = 0.735_DA_ + 10.5 (*R* ^2^ = 0.999) for TNW-P, *I*_pa_ = 0.503_DA_ + 6.57 (*R* ^2^ = 0.999) for TNW-C, and *I*_pa_ = 0.625_DA_ + 9.61 (*R* ^2^ = 0.999) for TNW-HRP.

**Table 3:** Determination of DA in Pharmaceutical Formulations Using the Comparatives Methods and the Biosensor Proposed (*n* = 3)^a^

DA (mg mL^–1^)			Relative error (%)	
Label value	Comparatives methods	TNW-POX biosensor	RE_1_	RE_2_
5.00	UV: 4.97 ± 0.04	5.04 ± 0.11	+1.2	+1.8 (UV)
				–9.8 (HPLC)
		TNW-P biosensor		
		5.47 ± 0.09	+9.4	+10 (UV)
				–2.1 (HPLC)
	HPLC: 5.59 ± 0.0002	TNW-C biosensor		
		4.99 ± 0.40	–0.2	+0.4 (UV)
				–10.7 (HPLC)
		TNW-HRP biosensor		
		4.99 ± 0.22	–0.2	+0.4 (UV)
				–10.7 (HPLC)

a Confidence level of 95%. RE_1_ = biosensor versus label value. RE_2_ = biosensor versus comparative method.

The TNW-P biosensor produced measurements exceeding the acceptable error threshold of 5%, restricting its accuracy to concentrations near the nominal value (5 mg mL^–1^). In contrast, the TNW-C biosensor achieved a relative error of only −0.2% compared to the labeled value, indicating excellent analytical performance. This result may be attributed to the greater diversity of natural components present in the extract, which can confer increased enzyme stability. Similarly, the TNW-HRP biosensor demonstrated strong analytical performance, with a relative error of −0.2% relative to the labeled value. A statistical analysis (mean ± SD, *n* = 3) showed that TNW-POX, TNW-C, and TNW-HRP biosensors did not differ significantly from the UV–vis spectrophotometry method (Welch’s *t*-test, *p* > 0.05). In contrast, TNW-P differed significantly (*p* = 0.004) from this method, and from the labeled value of 5.00 mg mL^–1^ (one-sample *t*-test, *p* = 0.012). Other methods showed no significant difference from the labeled value (*p* > 0.05).

The HPLC method measured a DA concentration of 5.59 ± 0.0002 mg mL^–1^, corresponding to a relative error of 11.8% compared with the labeled value. Although the chromatographic injections showed high instrumental repeatability, statistical inference was not performed because the measurements were obtained from instrumental duplicates rather than independent sample preparations. The HPLC result was higher than that obtained by UV–vis spectrophotometry (4.97 ± 0.06 mg mL^–1^). This difference may reflect methodological factors, including calibration matrix, dilution procedure, chromatographic response, or the detection of dopamine-related species under the selected analytical conditions. Therefore, although HPLC provides analyte separation prior to detection and is generally less susceptible to spectral interference than UV–vis spectrophotometry, further validation using independent sample preparations would be required before attributing this difference solely to improved selectivity. Overall, the TNW-POX, TNW-C, and TNW-HRP biosensors, as well as the UV–vis method, produced results closer to the labeled value than HPLC under the conditions evaluated.

## 4 Conclusion

Covalent immobilization of a peroxidase-rich catalytic extract derived from *Cucumis anguria* L. onto functionalized TNWs represents a practical and robust approach for constructing electrochemical biosensors, as demonstrated by electrochemical analyses. The new use of *Cucumis anguria* L. as a natural and sustainable peroxidase source enabled the fabrication of CPE biosensors for DA detection, exhibiting linear responses over the concentration range of 5–65.4 μmol L^–1^. Among the systems evaluated, the biosensor constructed with concentrated gherkin extract (TNW-C) demonstrated the best analytical performance, achieving a detection limit of 0.400 μmol L^–1^ and a sensitivity of 8.19 μA μmol L^–1^ cm^–2^. These values are comparable to, and in some cases exceed, those obtained with commercial HRP.

The biosensors demonstrated adequate selectivity, with minimal interference from urea and well-resolved oxidation peaks for DA and UA, supporting their applicability in complex biological matrices. Accurate quantification of DA in commercial pharmaceutical samples (4.99 ± 0.40 mg mL^–1^ for TNW-C, with a relative error of −0.2%) further validates the practical feasibility of this biosensing platform for real-sample analysis at concentrations relevant to plasma and urinary levels. Real-sample analysis in a commercial pharmaceutical formulation confirmed the practical performance of the proposed platform. TNW-C quantified DA as 4.99 ± 0.40 mg mL^–1^, corresponding to a relative error of −0.2% compared with the labeled value and showing agreement with UV–vis spectrophotometry. Although HPLC showed high instrumental repeatability, it yielded a higher concentration under the selected conditions, highlighting the importance of matrix-matched validation in comparative analyses.

Beyond DA detection, this study presents a broadly applicable strategy for substituting purified commercial enzymes with rationally processed plant extracts in electrochemical biosensors. Combined with nanostructured supports such as TNWs, these extracts provide a sustainable, viable alternative for next-generation biosensing devices, enabling economical, eco-friendly sensor fabrication. Future investigations should focus on optimizing extract purification, expanding the spectrum of phenolic analytes, and incorporating this platform into diverse electrochemical architectures, including screen-printed and three-dimensional electrode systems. Although the results demonstrate promising performance, several limitations persist. Plant-based biosensors encounter challenges related to extract variability, reproducibility, and long-term standardization. For future research, explicitly conduct expanded interference studies covering the entire range of basal physiological concentrations. Prioritize purified plant extracts over crude preparations to reduce variability. By taking these focused steps, researchers can drive the development of more reliable plant-based biosensors and significantly advance the field.

## Supplementary Material



## Notes

Additional characterization and analytical data: X-ray diffraction (XRD) patterns; energy-dispersive X-ray spectroscopy (EDX) spectra; optimization of experimental parameters for biosensor performance; interference studies; calibration plots obtained by UV–visible spectroscopy and high-performance liquid chromatography (HPLC); standard addition method results (PDF)
