# Supplementary material for: Electrochemical Dopamine Biosensor Based on Plant-Derived Peroxidase Immobilized on Titanate Nanowires
Source: ACS Omega. 2026 Jun 17;11(25):37801–17. doi: 10.1021/acsomega.6c02913 (PMC13325118; doi:10.1021/acsomega.6c02913)
Supplement: Supplementary file 1 [file ao6c02913_si_001.pdf]

Supporting Information

Electrochemical Dopamine Biosensor Based on Plant-Derived Peroxidase Immobilized on Titanate Nanowires

Daniel Ananias Reis de Campos, Guilherme Sales da Rocha*, Neuman Solange de Resende, Helen Conceição Ferraz, Inês Rosane Welter Zwirtes de Oliveira, João Victor Nicolini*

AUTHOR INFORMATION

Corresponding Authors

Guilherme Sales da Rocha − *Universidade Federal do Rio de Janeiro, Rio de Janeiro, 21941-853, State of Rio de Janeiro, BR*
Email: gsrocha@peq.coppe.ufrj.br
João Victor Nicolini − *Universidade Federal Rural do Rio de Janeiro, Seropédica, 23890-000, State of Rio de Janeiro, BR*
Email: jvnicolini@ufrrj.br

Authors

Daniel Ananias Reis de Campos − *Universidade Federal Rural do Rio de Janeiro, Seropédica, 23890-000, State of Rio de Janeiro, BR*
Neuman Solange de Resende − *Universidade Federal do Rio de Janeiro, Rio de Janeiro, 21941-853, State of Rio de Janeiro, BR*
Helen Conceição Ferraz − *Universidade Federal do Rio de Janeiro, Rio de Janeiro, 21941-853, State of Rio de Janeiro, BR*
Inês Rosane Welter Zwirtes de Oliveira − *Universidade Federal Rural do Rio de Janeiro, Seropédica, 23890-000, State of Rio de Janeiro, BR*

X-Ray Diffraction

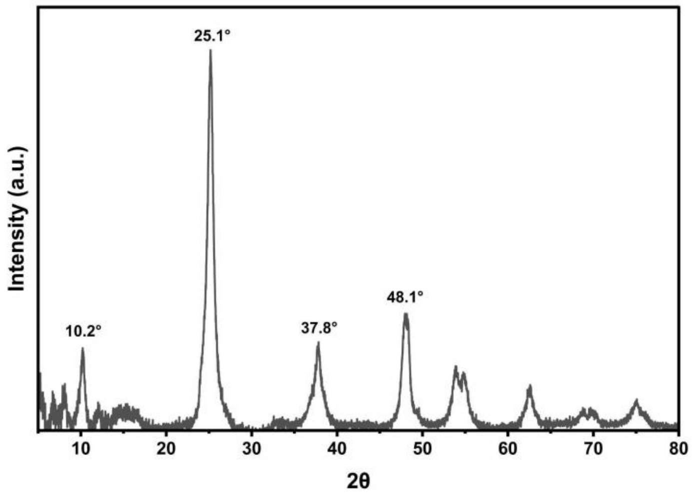

Figure S1. XRD patterns of the TNWs.

EDS/Elemental mapping

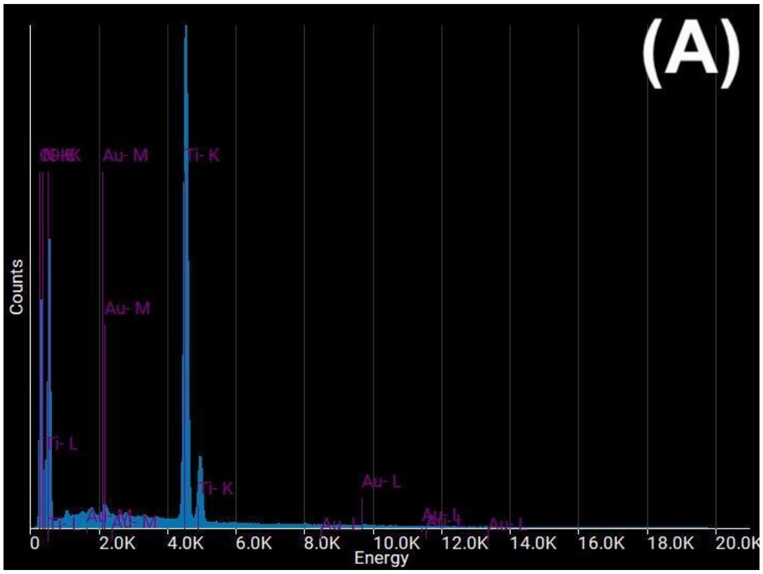

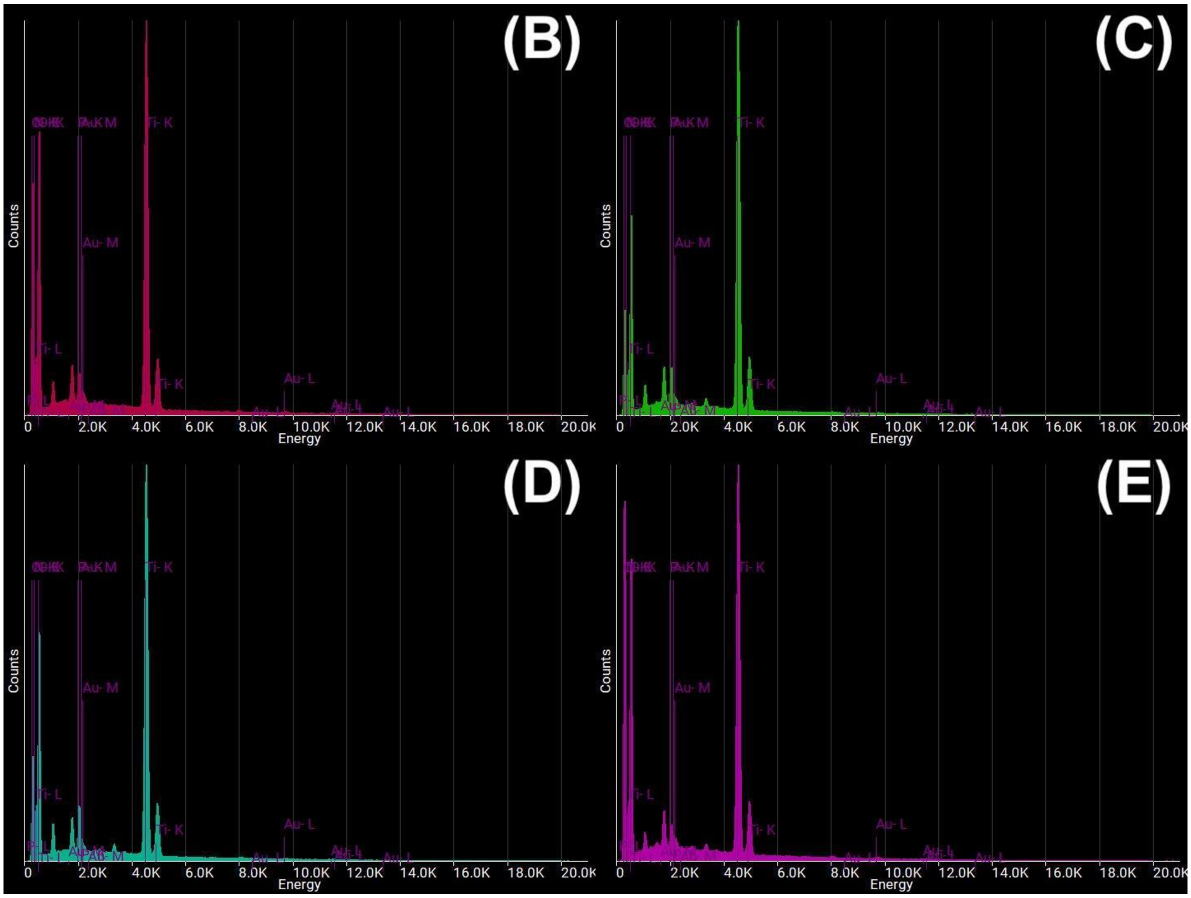

Figure S2. EDS spectrums: (A) TNW, (B) TNW-APTMS-GA, (C) TNW-POX, (D) TNW-C, and (E) TNW-P.

Experimental Parameters on Biosensor Performance

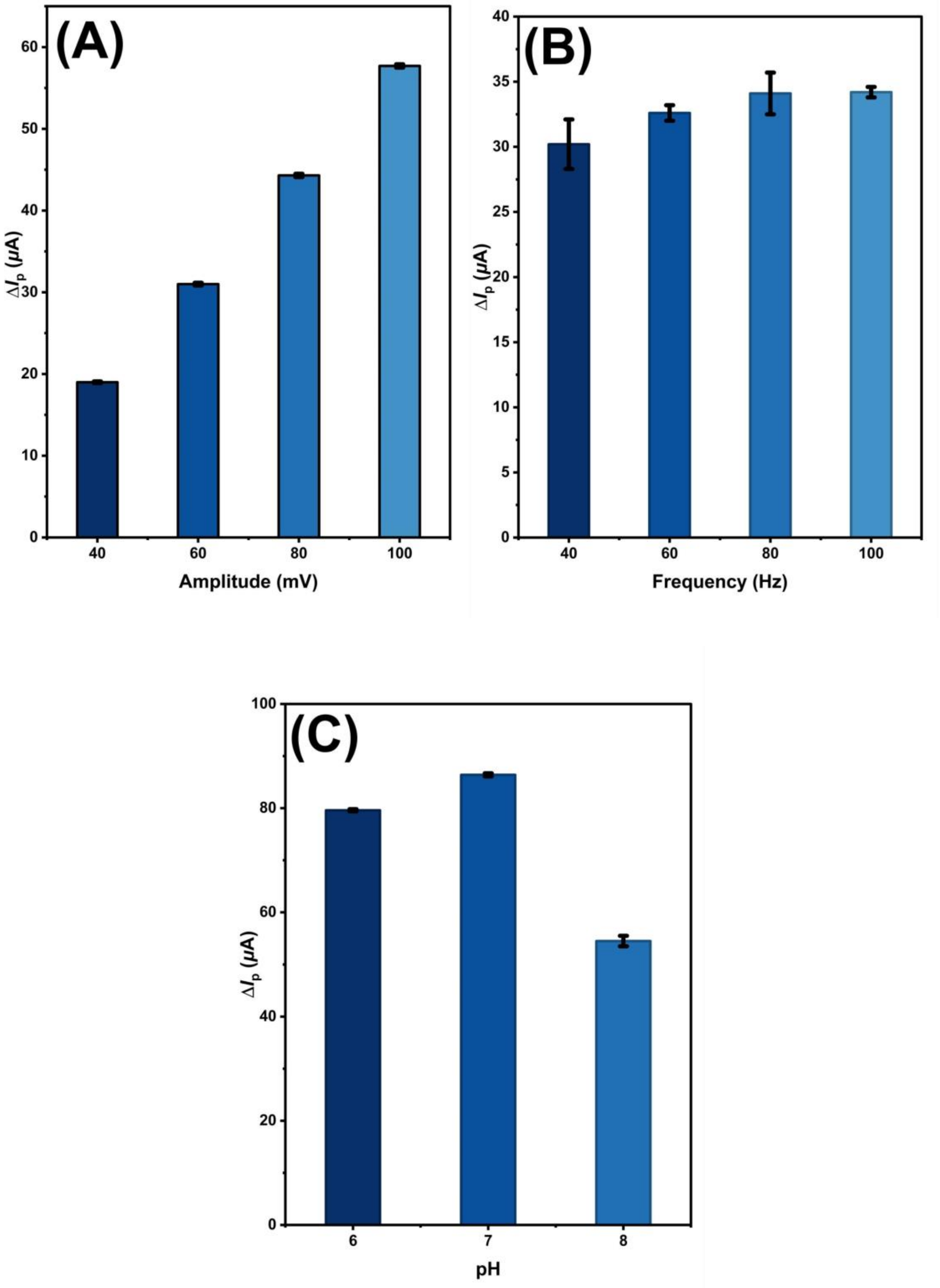

Figure S3. Experimental Parameters of the four proposed biosensors (Dopamine solution 0.2 mmol L^{-1} and H_2O_2 2 mmol L^{-1}). (A) Study of the effect of SWV at different amplitude values using biosensors in PBS pH 7, frequency of

100 Hz, and potential increment of 5 mV. (B) Study of the effect of SWV at different frequency values using biosensors in PBS pH 7, amplitude of 100 mV, and potential increment of 5 mV. (C) Study of the effect of SWV at different buffer solution pH values using biosensors, frequency of 100 Hz, amplitude of 100 mV and potential increment of 5 mV.

Interference test

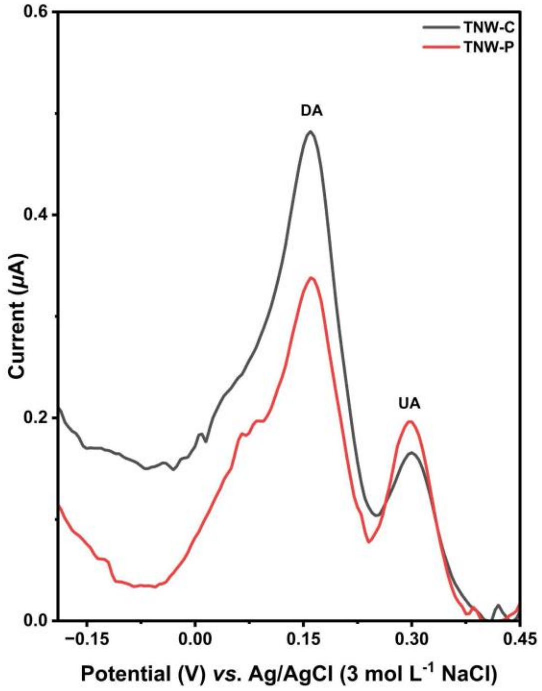

Figure S4. Interference analysis of TNW-C and TNW-P biosensors for DA detection at 10 μmol L^{-1} in the presence of 10 μmol L^{-1} UA using DPV.

Calibration plot (UV-Visible and High Performance Liquid Chromatography)

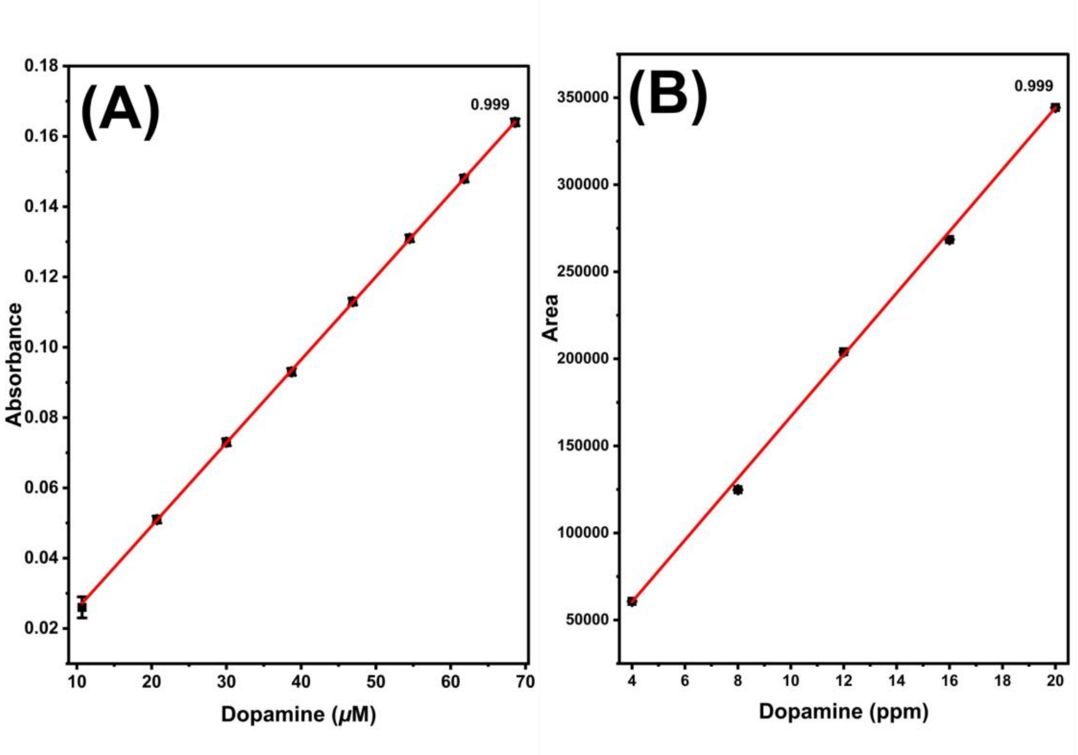

Figure S5. Analytical curve of (A) Dopamine concentration versus absorbance obtained by UV-Visible and (B) Dopamine concentration versus chromatographic peak area obtained by High Performance Liquid Chromatography.

Standard addition method

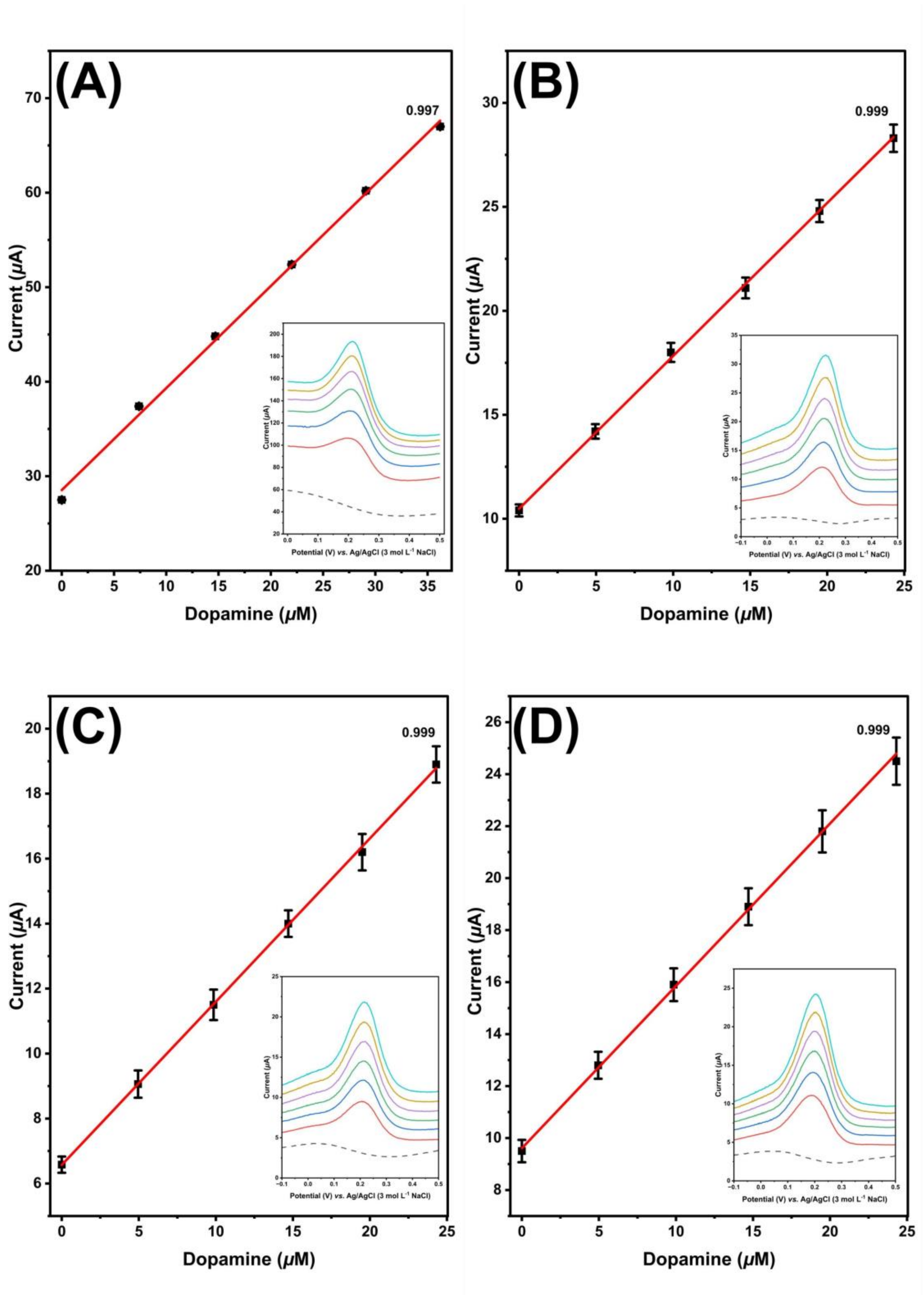

Figure S6. Analytical curve of standard addition in a commercial sample using (A) TNW-POX biosensor, (B) TNW-P biosensor, (C) TNW-C biosensor, and (D) TNW-HRP biosensor (n = 3).
